# Autologous cutis and subcutis micrografts combined with a collagen–elastin dermal scaffold for chronic wounds: a retrospective single-arm clinical study with prospectively collected data

**DOI:** 10.3389/fphar.2026.1891667

**Published:** 2026-07-31

**Authors:** Orestis Ioannidis, Konstantinos Zapsalis, Aggeliki Cheva, Elisavet Anestiadou, Panagiotis Savvidis, Nikoleta Pasteli, Ekaterini Klonou, Eirini Gemousakaki, Savvas Simeonidis, Stefanos Bitsianis, Efstathios Kotidis, Manousos George Pramateftakis, Ioannis Mantzoros, Stamatios Angelopoulos

**Affiliations:** 1 4th Department of Surgery, School of Medicine, Faculty of Health Sciences, General Hospital of Thessaloniki “G. Papanikolaou”, Aristotle University of Thessaloniki, Thessaloniki, Greece; 2 Pathology Department, Faculty of Medicine, Aristotle University of Thessaloniki, Thessaloniki, Greece; 3 Department of Pathology, “G. Papanikolaou” General Hospital, Thessaloniki, Greece

**Keywords:** chronic wounds, collagen–elastin scaffold, C-reactive protein, cutis and subcutis micrografts, dermal regeneration, patient-reported outcomes, regenerative medicine, wound healing

## Abstract

**Background:**

Chronic wounds remain a significant clinical challenge, characterised by sustained inflammation, impaired angiogenesis, and defective extracellular matrix remodelling. Autologous cutis and subcutis micrografts and collagen–elastin dermal scaffolds have each shown promise in wound healing, but their combination has not been previously investigated in a clinical study. This study aimed to evaluate the feasibility, safety, and preliminary effectiveness of autologous cutis and subcutis micrografts seeded onto a collagen–elastin dermal scaffold for the treatment of chronic wounds.

**Methods:**

A single-arm, single-centre, retrospective analysis of prospectively collected data was conducted. Forty-four patients with chronic wounds were screened; 34 met the eligibility criteria and received the intervention. Following surgical debridement, autologous cutis and subcutis tissue was harvested by punch biopsies and mechanically disaggregated to produce a micrograft suspension, which was used to imbibe a collagen–elastin dermal scaffold before application to the wound bed. The primary outcome was percentage reduction in wound surface area at day 90, measured by digital planimetry. Secondary outcomes included complete wound closure rate, Bates–Jensen Wound Assessment Tool (BWAT) scores, wound bed tissue composition, patient-reported outcomes (pain Visual Analogue Scale, Wound-QoL, EQ-5D-5L), systemic inflammatory markers (C-reactive protein, erythrocyte sedimentation rate, neutrophil-to-lymphocyte ratio), nutritional markers (albumin, prealbumin), and adverse events. Assessments were performed at baseline and at days 7, 14, 30, 60, and 90.

**Results:**

Thirty-four patients (mean age 62.8 ± 11.6 years; 58.8% male) were treated. Wound aetiologies included venous (35.3%), diabetic (26.5%), mixed arteriovenous (17.6%), pressure (11.8%), and post-surgical (8.8%) ulcers. The mean baseline wound surface area was 13.2 ± 9.1 cm^2^ with a median wound duration of 18 weeks. Thirty-two patients (94.1%) completed the 90-day follow-up. The mean wound surface area decreased from 13.2 ± 9.1 cm^2^ to 2.8 ± 3.6 cm^2^, corresponding to a mean reduction of 78.8% (95% CI, 71.4%–86.2%; p < 0.001). Complete wound closure was achieved in 21 of 34 patients (61.8%) by day 90, with a median time to closure of 71 days. Bates–Jensen Wound Assessment Tool scores improved from 37.8 ± 6.4 to 18.0 ± 6.6 (p < 0.001). Healthy granulation tissue increased from 31.8% to 90.6% of the wound bed (p < 0.001). Resting pain Visual Analogue Scale decreased from 5.8 ± 2.4 to 1.9 ± 1.6 (p < 0.001). Wound-QoL global score improved from 2.42 ± 0.74 to 0.88 ± 0.56 (p < 0.001), exceeding the minimal important difference. EQ-5D-5L index value improved from 0.56 ± 0.19 to 0.78 ± 0.15 (p < 0.001). Serum CRP decreased from 19.2 ± 13.4 to 5.8 ± 4.4 mg/L (p < 0.001), and albumin increased from 3.22 ± 0.50 to 3.74 ± 0.40 g/dL (p < 0.001). Fourteen adverse events occurred in 11 patients (32.4%); all were mild. No serious adverse events, hospitalisations, or amputations were recorded.

**Conclusion:**

The combination of autologous cutis and subcutis micrografts with a collagen–elastin dermal scaffold is a feasible and safe regenerative approach for chronic wounds, associated with substantial wound area reduction, high rates of complete closure, significant improvements in wound status, systemic inflammatory and nutritional markers, and patient-reported quality of life. Because the study lacked a concurrent control group, these results should be interpreted as evidence of feasibility and safety rather than of efficacy, and require confirmation in an adequately powered randomised controlled trial.

## 1 Introduction

Chronic wounds remain a major clinical and socioeconomic challenge, as they frequently fail to progress through the orderly phases of healing and instead become trapped in a prolonged inflammatory state. This impaired healing trajectory is associated with persistent tissue breakdown, increased susceptibility to infection, recurrent healthcare utilization, and marked deterioration in patient quality of life. The burden of chronic wounds is substantial; in a UK National Health Service analysis, annual wound-management costs were estimated at £8.3 billion in 2017/2018 (Guest et al., 2020; Frykberg and Banks, 2015).

The pathophysiology of chronic wounds is complex and multifactorial. Non-healing wounds are characterized by sustained inflammation, dysregulated macrophage activity, impaired angiogenesis, excessive protease activity, defective extracellular matrix remodeling, and inadequate re-epithelialization. Rather than transitioning effectively from inflammation to proliferation and remodeling, these wounds remain biologically stalled. This hostile microenvironment limits the capacity of resident and recruited cells to regenerate tissue and often compromises the effectiveness of conventional wound care alone (Frykberg and Banks, 2015).

Among emerging regenerative strategies, autologous cutis and subcutis micrografts have gained increasing attention as a point-of-care therapeutic option. Mechanical disaggregation of the harvested cutis and subcutis yields a suspension containing viable cellular and extracellular matrix microfragments with reparative potential, without the need for *ex vivo* culture expansion. The therapeutic activity of these micrografts is thought to be largely pharmacological in nature, mediated not by durable engraftment but by the local release of biologically active agents. Dermis-derived stromal and progenitor cells, together with the co-transplanted native extracellular matrix, secrete a broad repertoire of growth factors and cytokines—including vascular endothelial growth factor (VEGF), hepatocyte growth factor (HGF), transforming growth factor-β (TGF-β), basic fibroblast growth factor (bFGF), and interleukins—as well as extracellular vesicles carrying regulatory microRNAs (Baglioni et al., 2024; Sbitan et al., 2025). These mediators act on resident and recruited cells through paracrine signalling to promote angiogenesis, modulate inflammation, and stimulate cell migration and proliferation, effectively constituting a non-traditional, autologous biological pharmacotherapy delivered directly to the wound bed. In a recent pilot clinical study of chronic wounds refractory to conventional treatment, autologous micrograft therapy was associated with substantial wound area reduction and complete healing in a considerable proportion of patients by day 90 (Baglioni et al., 2024).

In parallel, acellular dermal scaffolds, particularly collagen-based or collagen–elastin matrices, have been increasingly used in reconstructive and wound surgery to provide a structural template for tissue regeneration. The matrix used in the present study is a non-cross-linked, acellular dermal template composed of native type I collagen and elastin, with a porous three-dimensional architecture that mimics the composition of healthy dermis. Beyond their purely mechanical scaffolding function, such matrices act as a provisional extracellular matrix that supports cellular infiltration, neovascularization, and dermal regeneration while improving the quality and organization of the healing tissue. Importantly, the collagen and elastin components are not biologically inert: on enzymatic degradation they release matrikines and elastin-derived peptides that can modulate fibroblast and endothelial cell behaviour, while the elastin fraction has been associated with reduced myofibroblast differentiation and improved, less fibrotic dermal remodelling. Human studies have shown that collagen–elastin dermal matrices can be beneficial in complex wounds and diabetic foot ulcers, including improved healing-related outcomes when used in conjunction with skin grafting (Jeon et al., 2013).

From a biological standpoint, the combination of autologous cutis and subcutis micrografts with a dermal scaffold appears especially attractive. The micrografts may provide a regenerative cellular and signaling stimulus, whereas the scaffold may provide the structural microenvironment necessary for cell retention, migration, neovascularization, and tissue organization. In theory, this combination may address both the cellular and extracellular deficiencies of chronic wounds and thereby enhance repair. However, the published human literature appears to include either adipose-derived stromal cells seeded onto a collagen matrix or separate studies of dermal scaffolds in wound reconstruction, rather than the specific clinical combination investigated here (Jeon et al., 2013; Lafosse et al., 2015).

Therefore, to the best of our knowledge, this is the first human clinical study to investigate the combination of autologous cutis and subcutis micrografts with a collagen–elastin dermal scaffold for the treatment of chronic wounds. Because the current literature is limited and largely based on small observational cohorts, there remains a clear need for prospective clinical studies evaluating not only wound closure outcomes but also the early biological changes induced within the wound bed (Baglioni et al., 2024; Lafosse et al., 2015).

The present study was therefore conducted as a retrospective, single-arm, single-center analysis of prospectively collected data to evaluate the feasibility, safety, and preliminary effectiveness of treating chronic wounds with autologous cutis and subcutis micrografts in combination with a collagen–elastin dermal scaffold, using clinical, patient-reported, and systemic biomarker outcomes (Frykberg and Banks, 2015; Baglioni et al., 2024).

## 2 Methods

### 2.1 Study design and setting

This was a single-arm, single-centre, retrospective analysis of prospectively collected data, designed to evaluate the feasibility, safety, and preliminary effectiveness of treating chronic wounds with autologous cutis and subcutis micrografts in combination with a collagen–elastin dermal scaffold. The study was conducted at George Papanikolaou University Hospital, Thessaloniki, Greece, between 8/2/2025 and 20/2/2026. The study has received approval from the Scientific Board of G. Papanikolaou University Hospital under protocol number 30.04.2026/267, and it was conducted in accordance with the principles of the Declaration of Helsinki (2013 revision) (World Medical Association, 2013). All participants had provided written informed consent for their treatment and for the use of their clinical data prior to undergoing the procedure; institutional approval for the retrospective analysis of these data was subsequently obtained (see Section 2.9). All clinical data were collected prospectively during routine clinical care, using a standardised assessment protocol with predefined outcome measures and follow-up time points, and were subsequently analysed retrospectively for the purposes of this study; no aspect of patient management was altered for research purposes.

### 2.2 Participants

#### 2.2.1 Eligibility criteria

Adult patients (aged ≥18 years) presenting with chronic wounds were screened for eligibility. For the purposes of this study, a chronic wound was defined as a wound of any aetiology that had failed to proceed through the orderly stages of healing and had not demonstrated significant progress toward closure despite at least 4–6 weeks of appropriate standard wound care (Frykberg and Banks, 2015). Inclusion criteria were as follows: (1) presence of a chronic wound as defined above; (2) wound surface area of at least 2 cm^2^ at the time of screening; (3) adequate nutritional status (serum albumin ≥2.5 g/dL); (4) willingness and ability to attend all scheduled follow-up visits; and (5) provision of written informed consent.

Exclusion criteria were as follows: (1) clinical evidence of active, untreated wound infection at the time of intervention; (2) known hypersensitivity to bovine-derived collagen or elastin products; (3) uncontrolled diabetes mellitus (haemoglobin A1c >12%); (4) current use of systemic immunosuppressive therapy, chemotherapy, or systemic corticosteroids at doses exceeding 10 mg/day of prednisolone or equivalent; (5) known active malignancy; (6) significant peripheral arterial disease precluding wound healing (ankle–brachial pressure index<0.5, or clinical judgement indicating unsalvageable limb ischaemia); (7) pregnancy or breastfeeding; and (8) participation in another interventional clinical trial within the preceding 30 days.

### 2.3 Intervention

**FIGURE 1:**
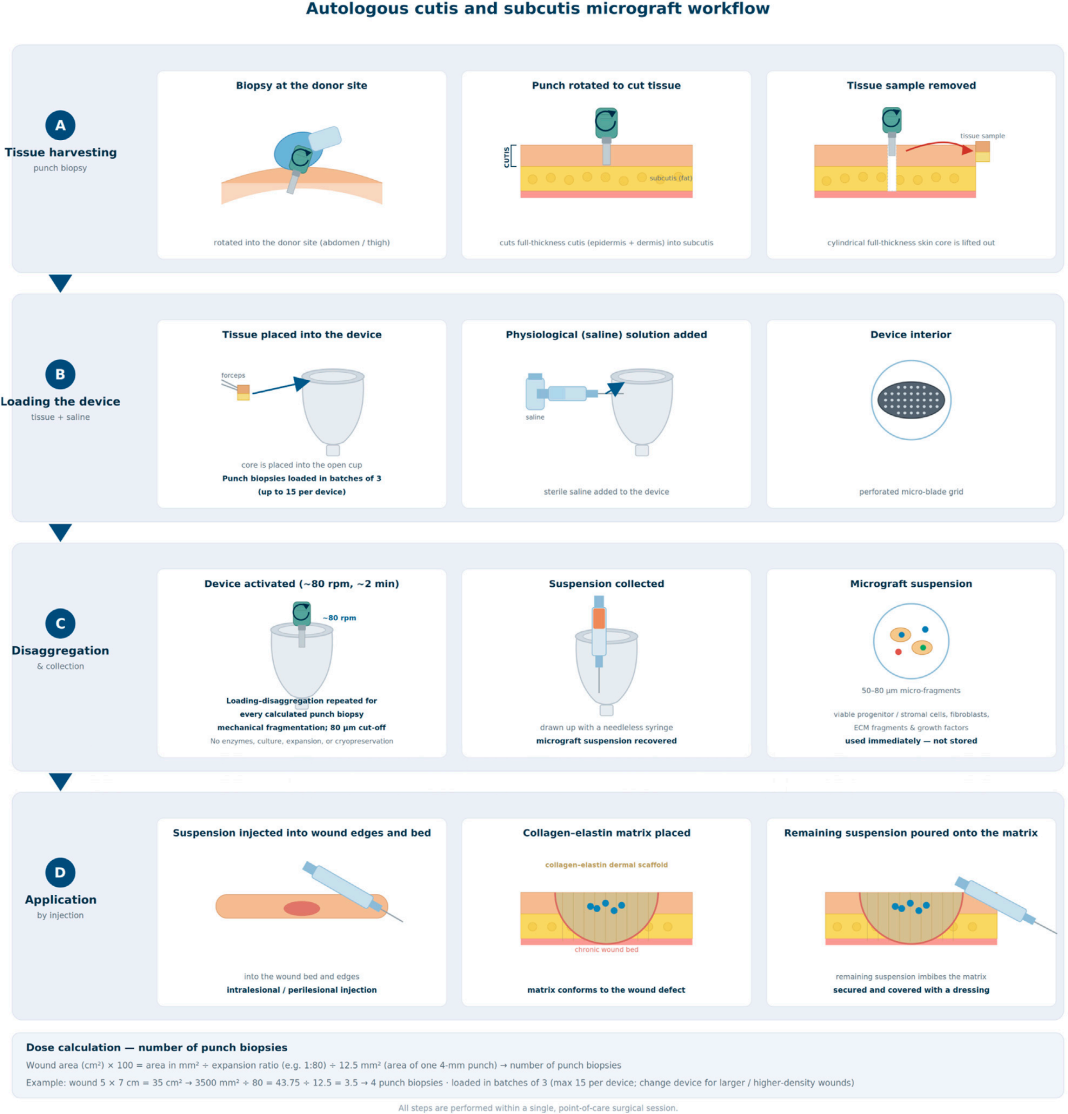
Overview of the autologous cutis and subcutis micrograft workflow. **(A)** Tissue harvesting: a skin punch biopsy is rotated into the donor site (abdomen or thigh) to cut and lift out a cylindrical full-thickness skin core comprising cutis (epidermis and dermis) and underlying subcutis (fat). **(B)** Loading the device: the harvested cores are placed into the disposable chamber together with sterile physiological (saline) solution and disaggregated against a perforated micro-blade grid; punch biopsies are loaded in batches of three (up to 15 per device). **(C)** Disaggregation and collection: the device is activated (∼80 rpm for ∼2 min) to mechanically fragment the tissue (80 µm cut-off; no enzymes, culture, expansion, or cryopreservation), and the resulting micrograft suspension—containing viable progenitor/stromal cells, fibroblasts, extracellular-matrix fragments, and growth factors—is drawn up with a needleless syringe and used immediately. **(D)** Application: the suspension is injected into the wound bed and edges (intralesional/perilesional), a collagen–elastin dermal scaffold is placed to conform to the wound defect, and the remaining suspension is poured onto the scaffold before a dressing is applied. The number of punch biopsies is calculated from the wound area and the desired expansion ratio (see inset formula). All steps are performed within a single, point-of-care surgical session.

An overview of the complete intervention—from autologous tissue harvesting through micrograft preparation and application—is presented in Figure 1.

#### 2.3.1 Wound bed preparation

Prior to the application of the study intervention, all wounds underwent thorough surgical debridement to remove devitalised tissue, slough, and biofilm, with the aim of establishing a clean, well-vascularised wound bed (Frykberg and Banks, 2015). Debridement was performed under local or regional anaesthesia as clinically appropriate. Following debridement, haemostasis was achieved and the wound was irrigated with sterile normal saline.

#### 2.3.2 Cutis and subcutis harvesting and micrograft preparation

**FIGURE 2:**
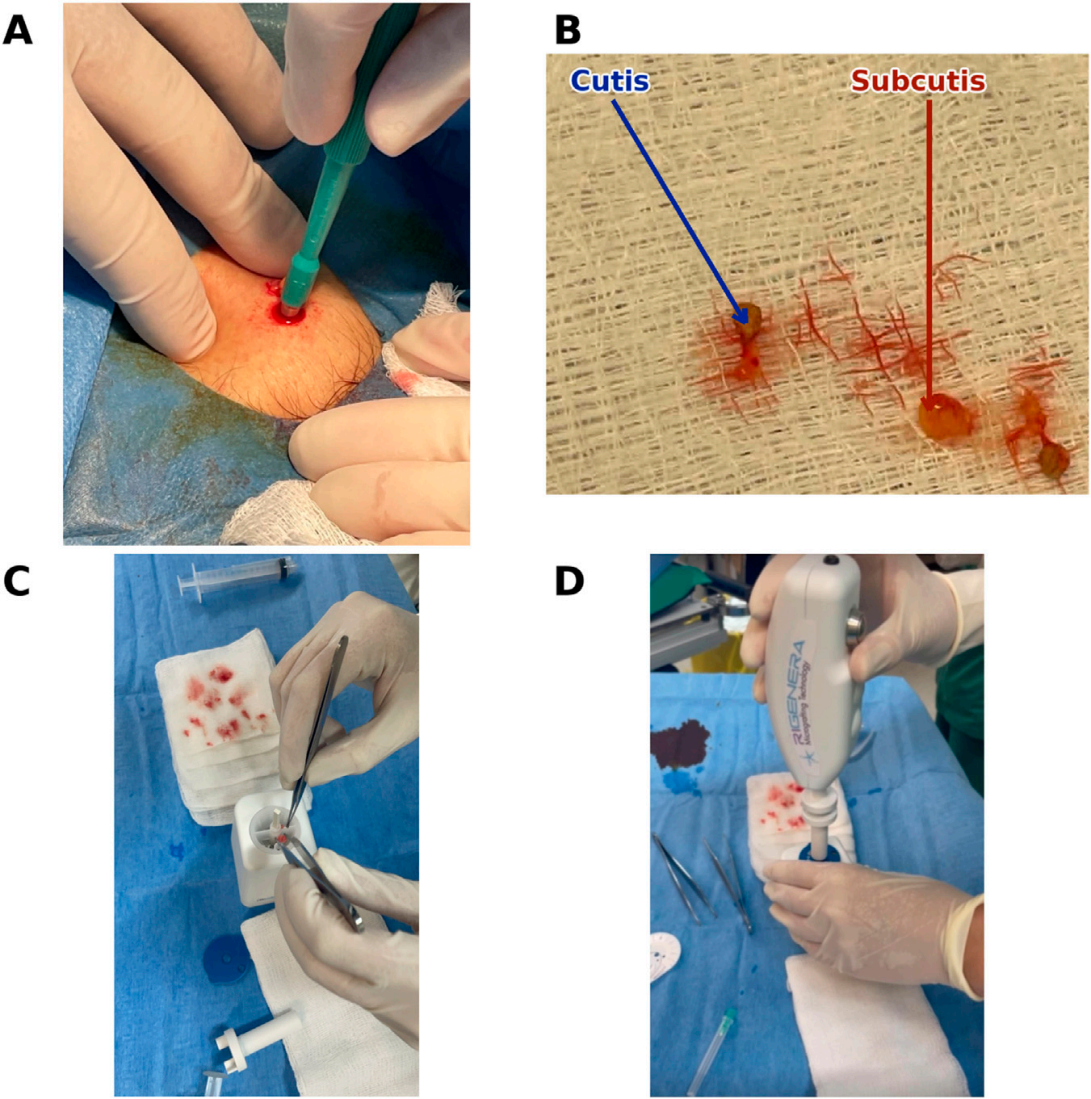
Key steps of autologous cutis and subcutis harvesting and micrograft preparation. **(A)** Harvesting of a full-thickness cutis and subcutis specimen from the donor site using a skin punch biopsy. **(B)** Harvested tissue cores on gauze; the arrows indicate the cutis (epidermis and dermis) and the underlying subcutis (subcutaneous adipose tissue). **(C)** Loading of the harvested tissue into the disposable chamber of the mechanical disaggregation device. **(D)** Mechanical disaggregation of the tissue with the micrografting device to produce the autologous micrograft suspension.

Autologous cutis and subcutis tissue was harvested from the abdominal region or the thigh of each patient under local anaesthesia using punch biopsies. These full-thickness punch biopsies inherently included the dermis together with an amount of underlying subcutaneous adipose (lipid) tissue, so the resulting micrografts comprised predominantly dermal tissue with an adipose component. This technique is most commonly described in the literature as “dermis micrografts” or “dermal micrografts” (De Francesco et al., 2017; Marcarelli et al., 2017; Baglioni et al., 2016; Tresoldi et al., 2019); the authors, however, consider “cutis and subcutis micrografts” to be a more anatomically accurate designation, since the punch biopsy harvests the full thickness of the cutis—comprising both the epidermis and the dermis—together with a portion of the underlying subcutis (subcutaneous adipose tissue). The harvested tissue was then processed by mechanical disaggregation using a CE-marked Class IIa medical device equipped with a grid of micro-blades and micro-pores, which selects tissue microfragments through a dimensional exclusion system (microfragments of approximately 50–80 µm) (Baglioni et al., 2024). The disaggregation was performed in a closed, sterile system at the point of care, within the same operative session and without interruption of the surgical procedure. No enzymatic digestion, centrifugation, cryopreservation, or *ex vivo* culture expansion was employed, and no ancillary reagents were added; processing therefore involved only mechanical manipulation. The resulting intermediate product was a fluid micrograft suspension containing viable autologous cells (cutis- and subcutis-derived stromal and progenitor cells, fibroblasts, other resident dermal cells, and a minor adipose-derived cellular fraction), intact extracellular matrix microfragments, and endogenous growth factors. In accordance with the manufacturer’s specifications and previous characterisation of comparable preparations, the suspension is expected to provide a high proportion of viable nucleated cells; where feasible, product quality was confirmed intra-operatively by macroscopic inspection of a homogeneous suspension and absence of visible clots. The suspension was used immediately and was not stored, minimising any reduction in cell viability (Baglioni et al., 2024; Sbitan et al., 2025). The key steps of cutis and subcutis harvesting and micrograft preparation are illustrated in Figure 2.

#### 2.3.3 Collagen–elastin dermal scaffold preparation and application

The dermal scaffold used was a non-cross-linked, acellular dermal matrix composed of native type I collagen and elastin with a porous three-dimensional architecture. The matrix was cut to the dimensions of the wound and rehydrated with sterile normal saline in accordance with the manufacturer’s instructions (Jeon et al., 2013). The micrograft suspension was applied in two stages to match the operative sequence: a portion of the suspension was first applied directly to the prepared wound bed, the rehydrated collagen–elastin scaffold was then positioned over the wound bed, and the remaining suspension was applied onto the scaffold to imbibe the matrix, ensuring uniform distribution of the autologous cellular and extracellular material both beneath and throughout the scaffold. The scaffold was secured in place using adhesive strips, and an appropriate secondary dressing was applied over it. The rationale for combining autologous micrografts with this dermal scaffold was to deliver both a regenerative cellular and paracrine stimulus and a structural microenvironment that retains the micrografts within the wound, protects the cellular and extracellular components, and provides physical guidance for cell migration, neovascularisation, and tissue organisation; the collagen–elastin matrix may additionally exert a biologically active role through release of matrikines and elastin-derived peptides rather than serving a purely mechanical function (Baglioni et al., 2024; Jeon et al., 2013; Lafosse et al., 2015).

#### 2.3.4 Postoperative care

Postoperative wound care followed a standardised protocol. The initial dressing was left undisturbed for 3 days, after which the wound was inspected and redressed at intervals of 3–5 days using the same dressing regimen. Off-loading, compression therapy, or other adjunctive measures were employed as clinically indicated based on wound aetiology. No additional regenerative therapies (e.g., platelet-rich plasma, growth factor application, or negative-pressure wound therapy) were administered during the study period. Systemic antibiotics were prescribed only when clinical signs of wound infection were present and were documented.

#### 2.3.5 Regulatory classification of the cellular product

The autologous cutis and subcutis micrografts used in this study were prepared by mechanical disaggregation only, without enzymatic digestion, expansion, or culture, and were therefore regarded as a minimally manipulated cellular product. The micrografts were applied for a homologous (structural and reparative) purpose, within the same surgical procedure in which the tissue was harvested. Under the European Union framework governing human cells and tissues—principally Directive 2004/23/EC, together with Regulation (EC) No 1394/2007 on advanced therapy medicinal products (ATMPs)—minimally manipulated autologous tissue used for homologous purposes within a single point-of-care surgical procedure is generally not classified as an ATMP and falls outside the scope of centralised ATMP authorisation. The disaggregation device and the collagen–elastin matrix were both CE-marked medical devices used in accordance with their intended purpose, and all tissue handling complied with applicable national and institutional requirements. No cells were stored, banked, cryopreserved, or transferred between procedures.

### 2.4 Outcome measures

All outcome assessments were performed at baseline (day 0, immediately prior to the intervention) and at predefined follow-up time points: days 7, 14, 30, 60, and 90 post intervention. Additional assessments beyond day 90 were recorded where available. Clinical assessments were performed independently by two trained investigators using standardised protocols; where assessments differed, the recorded value was reached by consensus, and inter-observer agreement was monitored to minimise variability.

#### 2.4.1 Primary outcome

The primary outcome was the percentage reduction in wound surface area from baseline to day 90. Wound surface area was measured at each study visit using standardised digital photography and digital planimetry (Wendelken et al., 2011; Foltynski et al., 2015). All wound photographs were obtained under consistent lighting conditions with the camera lens positioned perpendicular to the wound plane. Two parallel calibration rulers were placed at the superior and inferior wound margins to enable accurate scaling (Foltynski et al., 2015). Digital planimetry was performed using ImageJ (version 1.54; National Institutes of Health, Bethesda, MD, United States), with the wound perimeter traced manually on the calibrated digital image by a trained assessor blinded to the clinical timepoint where feasible. Wound surface area was calculated in square centimetres (cm^2^). This outcome is consistent with the primary endpoint reported in previous micrograft studies (Baglioni et al., 2024).

#### 2.4.2 Secondary clinical outcomes

Secondary clinical outcomes included the following:

Complete wound closure rate: defined as the proportion of wounds achieving full epithelialization, without drainage or need for dressings, confirmed at two consecutive study visits at least 14 days apart (Frykberg and Banks, 2015; Baglioni et al., 2024).

Time to complete wound closure: recorded as the number of days from the intervention (day 0) to the first visit at which complete wound closure was confirmed.

Wound bed tissue composition: the proportion of the wound bed occupied by healthy granulation tissue, slough, necrotic tissue, and epithelializing tissue was estimated visually by two independent trained assessors and recorded as a percentage at each study visit, with the mean of the two assessments used for analysis and discrepancies greater than 10% resolved by consensus. The percentage of healthy granulation tissue has been proposed as a valid intermediate endpoint in chronic wound trials (Enoch et al., 2004). Standardised wound photographs were used to corroborate clinical assessment.

Wound assessment scores: wound status was evaluated at each visit using the Bates–Jensen Wound Assessment Tool (BWAT) (Bates-Jensen et al., 1992; Bates-Jensen et al., 2019). The BWAT is a validated 15-item instrument that evaluates wound size, depth, edges, undermining, necrotic tissue type and amount, exudate type and amount, surrounding skin colour, peripheral tissue oedema and induration, granulation tissue, and epithelialization. Each of the 13 scored items is rated on a modified Likert scale (1 = least severe to 5 = most severe), with lower total scores indicating a more favourable wound status (Bates-Jensen et al., 1992). BWAT scores were plotted longitudinally to track wound healing trajectories.

Wound exudate: the type (serous, serosanguineous, sanguineous, or purulent) and amount (none, scant, moderate, or heavy) of wound exudate were recorded at each visit. Perilesional skin status: the condition of the skin surrounding the wound was assessed at each visit and documented, including the presence or absence of erythema, maceration, oedema, induration, and pigmentation changes.

Wound infection: clinical signs of wound infection were assessed at each visit using established clinical criteria (increasing pain, spreading erythema, local warmth, oedema, purulent discharge, wound breakdown, or systemic signs such as pyrexia) (International Wound Infection Institute IWII, 2022). Wound swab cultures were obtained when clinically indicated. The use of systemic antibiotics during the study period was recorded.

#### 2.4.3 Patient-reported outcome measures

Patient-reported outcomes were assessed at baseline, day 30, and day 90 using the following validated instruments:

Wound pain: pain intensity was assessed at each study visit using a 10-cm Visual Analogue Scale (VAS), anchored at 0 (“no pain”) and 10 (“worst pain imaginable”) (Iglesias Lopez et al., 2023). Patients rated both resting pain and pain at dressing change. Pain reduction has been identified as a clinically meaningful outcome in regenerative wound therapies (Sbitan et al., 2025; Iglesias Lopez et al., 2023).

Disease-specific quality of life: health-related quality of life was assessed using the Wound-QoL questionnaire, a validated, wound-specific patient-reported outcome measure comprising 17 items across three subscales (body, psyche, and everyday life) (Topp et al., 2021; Blome et al., 2014). Each item is scored from 0 (not at all) to 4 (very much), and a global score is calculated, with higher scores indicating greater impairment. The Wound-QoL has demonstrated high internal consistency (Cronbach’s α > 0.8), good test–retest reliability, and satisfactory convergent validity with the EQ-5D (Topp et al., 2021).

Generic health-related quality of life: the EuroQol five-dimension five-level questionnaire (EQ-5D-5L) was administered to assess generic health status across five domains: mobility, self-care, usual activities, pain/discomfort, and anxiety/depression (Herdman et al., 2011). Each domain was scored on a five-level scale. The EQ Visual Analogue Scale (EQ- VAS) was also recorded, with patients rating their overall health on a vertical scale ranging from 0 (worst imaginable health state) to 100 (best imaginable health state). Both the EQ-5D and EQ-VAS have been used extensively in chronic wound populations (Herdman et al., 2011; Herber et al., 2007).

#### 2.4.4 Laboratory outcomes

Venous blood samples were obtained at baseline and at days 30 and 90 for the measurement of haematological, inflammatory, nutritional, and metabolic parameters. The following laboratory investigations were performed:

Inflammatory markers: serum C-reactive protein (CRP) was measured as a systemic indicator of the inflammatory state. CRP has been shown to be a sensitive and reliable marker reflecting the inflammatory burden of chronic wounds and to correlate with healing trajectory (Liu et al., 2014; Wu et al., 2025). Erythrocyte sedimentation rate (ESR) was measured as a complementary inflammatory marker.

Haematological parameters: a full blood count with differential white blood cell count was obtained. Parameters of interest included total white blood cell count (WBC), absolute neutrophil count, absolute lymphocyte count, the neutrophil-to-lymphocyte ratio (NLR) as a marker of systemic inflammation, haemoglobin concentration, haematocrit, and platelet count (Wu et al., 2025; Cervero-Ferragut et al., 2017).

Nutritional markers: serum albumin, serum prealbumin (transthyretin), and total protein were measured as indicators of nutritional and metabolic status. Hypoalbuminaemia has been associated with impaired wound healing outcomes and serves as a surrogate marker of chronic inflammation and catabolic state (Kim et al., 2023; Gau et al., 2024).

Glycaemic control: fasting blood glucose and glycated haemoglobin (HbA1c) were measured at baseline and at day 90 in all patients with diabetes mellitus to evaluate glycaemic control, given the established association between hyperglycaemia and impaired wound healing (Frykberg and Banks, 2015).

Renal function: serum creatinine and estimated glomerular filtration rate (eGFR) were recorded to characterise renal function, given its relevance to wound healing and nutritional status.

#### 2.4.5 Safety outcomes

Safety was assessed throughout the study period. All adverse events (AEs) and serious adverse events (SAEs) occurring from the date of the intervention through to the final study visit were recorded. Adverse events of particular interest included wound infection (both superficial and deep), wound dehiscence or graft failure, haematoma or seroma at the donor or recipient site, donor-site complications (pain, infection, delayed healing), unplanned hospitalisation related to the wound, need for re-intervention (including repeat debridement, skin grafting, or surgical revision), and amputation. The relationship of each adverse event to the study intervention was assessed by the investigator. Donor-site healing was also documented.

### 2.5 Data collection and documentation

Patient demographic and baseline clinical data were recorded at the time of enrolment, including age, sex, body mass index (BMI), relevant comorbidities (diabetes mellitus, peripheral vascular disease, chronic venous insufficiency, hypertension, immunosuppression, smoking status), wound aetiology, wound duration, wound location, number of wounds, and prior wound treatments. All data were recorded on standardised case report forms and entered into an electronic database.

#### 2.5.1 Schedule of assessments

Table 1 summarises the schedule of assessments performed at each study visit.

**TABLE 1:** Schedule of study assessments.

Assessment	Day 0	Day 7	Day 14	Day 30	Day 60	Day 90
Informed consent	✓	​	​	​	​	​
Demographics and history	✓	​	​	​	​	​
Wound photography and planimetry	✓	✓	✓	✓	✓	✓
Wound surface area (cm^2^)	✓	✓	✓	✓	✓	✓
BWAT score	✓	✓	✓	✓	✓	✓
Wound bed composition (%)	✓	✓	✓	✓	✓	✓
Exudate and perilesional skin	✓	✓	✓	✓	✓	✓
Pain VAS	✓	✓	✓	✓	✓	✓
Wound-QoL	✓	​	​	✓	​	✓
EQ-5D-5L	✓	​	​	✓	​	✓
CRP, ESR	✓	​	​	✓	​	✓
FBC with differential	✓	​	​	✓	​	✓
Albumin, prealbumin, total protein	✓	​	​	✓	​	✓
HbA1c, fasting glucose	✓	​	​	​	​	✓
Creatinine, eGFR	✓	​	​	​	​	✓
Wound swab (if indicated)	✓	✓	✓	✓	✓	✓
Adverse events	​	✓	✓	✓	✓	✓
Donor-site assessment	​	✓	✓	✓	✓	✓

BWAT, Bates–Jensen wound assessment tool; VAS, visual analogue scale; Wound-QoL, wound quality of life questionnaire; EQ-5D-5L, EuroQol five-dimension five-level questionnaire; CRP, C-reactive protein; ESR, erythrocyte sedimentation rate; FBC, full blood count; HbA1c, glycated haemoglobin; eGFR, estimated glomerular filtration rate.

### 2.6 Photographic documentation

Standardised wound photographs were obtained at every study visit. Images were captured using a digital single-lens reflex (D-SLR) camera under standardised and reproducible conditions, including consistent lighting, a fixed focal distance, and perpendicular camera orientation relative to the wound plane (Foltynski et al., 2015). Two parallel calibration rulers were placed in the same plane as the wound at its superior and inferior margins. A colour calibration card was included in the field of view to enable consistent colour reproduction across time points. Patient-identifying information was excluded from photographic images, and all images were assigned a unique coded identifier for data storage and analysis.

### 2.7 Sample size considerations

As this is an exploratory, single-arm feasibility study with no prior clinical data on the specific combination of autologous cutis and subcutis micrografts with a collagen–elastin dermal scaffold, a formal *a priori* sample size calculation based on a predefined effect size was not performed (Baglioni et al., 2024; Lafosse et al., 2015). Instead, a pragmatic sample of 40 consecutive patients meeting the eligibility criteria was planned. This sample size was considered sufficient to provide preliminary estimates of treatment effect size, variability, safety, and feasibility, and to inform the design and power calculation of a future controlled trial. The study is therefore not powered to demonstrate statistically significant efficacy and is intended to generate hypothesis-generating data.

### 2.8 Statistical analysis

All analyses were performed on a modified intention-to-treat (mITT) basis, including all patients who received the study intervention and attended at least one post-treatment follow-up visit; outcomes were analysed using observed (available-case) data without imputation for missing values arising from loss to follow-up. Statistical analyses were conducted using IBM SPSS Statistics (version 29.0; IBM Corp., Armonk, NY, United States).

Continuous variables were assessed for normality using the Shapiro–Wilk test and expressed as mean ± standard deviation (SD) for normally distributed data or as median and interquartile range (IQR) for non-normally distributed data. Categorical variables were expressed as absolute frequencies and percentages. Baseline demographic and clinical characteristics were summarised descriptively.

The primary outcome, percentage reduction in wound surface area, was calculated at each time point as: [(baseline area – area at time point)/baseline area] × 100. Changes in wound surface area and other continuous outcome measures from baseline to each follow-up time point were analysed using paired t-tests or Wilcoxon signed-rank tests, as appropriate. For variables measured at multiple time points, overall longitudinal trends were evaluated using the Friedman test, with pairwise comparisons versus baseline performed using the Wilcoxon signed-rank test.

Changes in BWAT scores, pain VAS, Wound-QoL scores, EQ-5D-5L index values, and EQ-VAS scores were analysed longitudinally using paired comparisons at each time point relative to baseline. Laboratory parameters (CRP, ESR, albumin, prealbumin, haemoglobin, HbA1c) were similarly compared between baseline and follow-up time points.

Complete wound closure was described as a cumulative proportion at each time point and presented using Kaplan–Meier survival analysis, with time to complete closure as the event. Patients who did not achieve wound closure by the final study visit were censored at the time of their last assessment.

Safety data were reported descriptively, including the frequency, type, severity, and relationship to treatment of all adverse events. A two-sided p-value of less than 0.05 was considered statistically significant for all analyses. Given the exploratory nature of the study, no adjustments for multiple comparisons were applied; however, all p-values are reported to enable interpretation in context.

### 2.9 Ethical considerations

The study has received approval from the Scientific Board of G. Papanikolaou University Hospital under protocol number 30.04.2026/267, and was conducted in full conformity with the Declaration of Helsinki (World Medical Association, 2013). This institutional approval covered the retrospective collection and analysis of the routinely acquired clinical data; because the patients had been managed according to standard clinical practice rather than a prospective research protocol, the approval was obtained after their treatment and follow-up had been completed. All participants provided written informed consent for treatment and for the use of their clinical data prior to undergoing the procedure. Patient data were handled in accordance with the General Data Protection Regulation (EU 2016/679), and all data were pseudonymised for analysis. The study has been reported in accordance with the Strengthening the Reporting of Observational Studies in Epidemiology (STROBE) statement (von Elm et al., 2007).

## 3 Results

### 3.1 Patient enrolment and baseline characteristics

**FIGURE 3:**
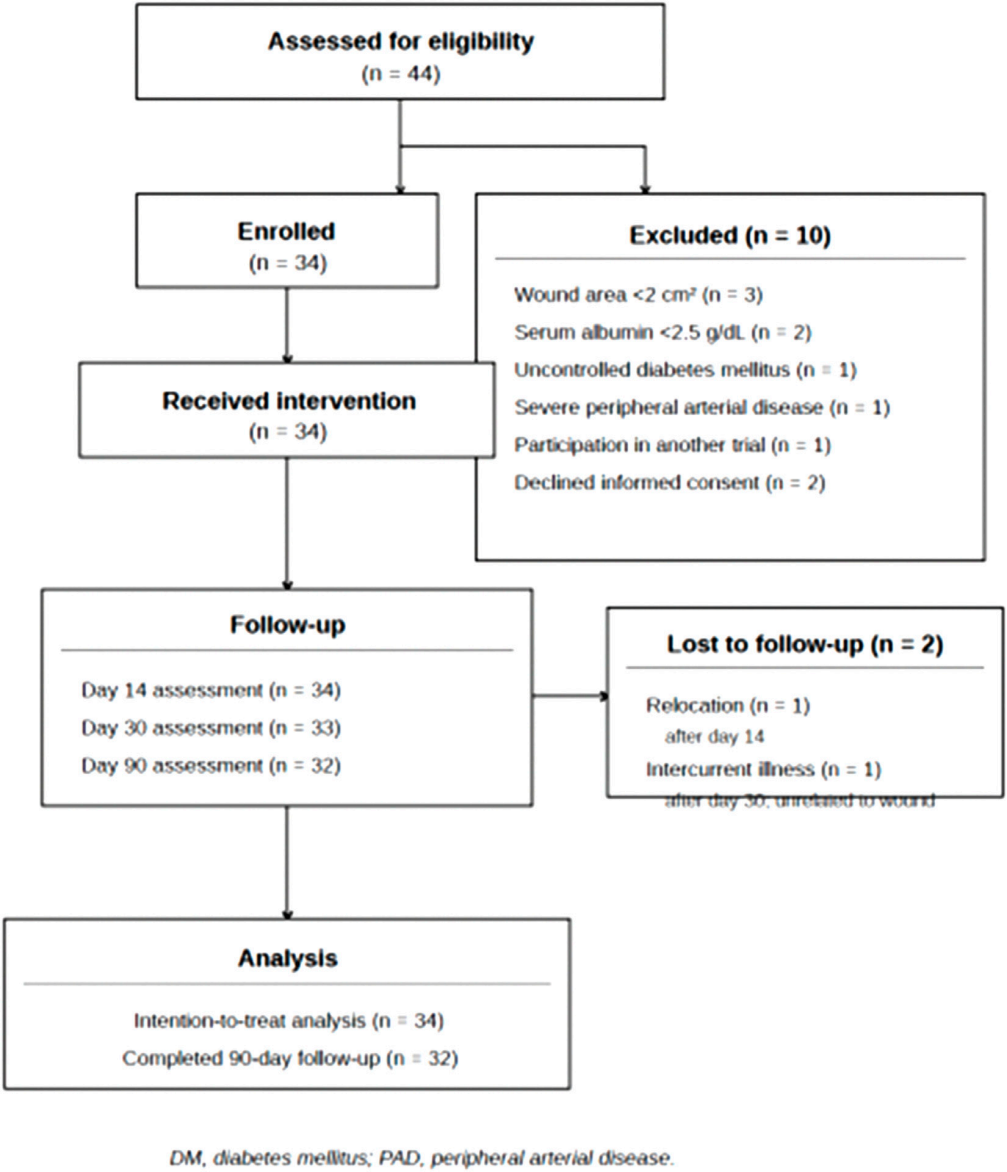
Patient flow diagram (STROBE). Forty-four patients screened; 10 excluded; 34 enrolled and treated; 32 completed 90-day follow-up.

Between 18/2/2025 and 20/2/2026, a total of 44 patients with chronic wounds were screened for eligibility. Ten patients were excluded: three had a wound surface area of less than 2 cm^2^, two had a serum albumin level below 2.5 g/dL, one had uncontrolled diabetes mellitus (HbA1c >12%), one had significant peripheral arterial disease (ankle–brachial pressure index <0.5), one was participating in another interventional trial, and two declined to provide informed consent. Thirty-four patients were enrolled and received the study intervention. Two patients were lost to follow-up (one after day 14 due to relocation and one after day 30 due to intercurrent illness unrelated to the wound). Thus, 34 patients were included in the modified intention-to-treat analysis and 32 patients completed the full 90-day follow-up (Figure 3).

Baseline demographic and clinical characteristics are presented in Table 2. The mean age was 62.8 ± 11.6 years, and 20 patients (58.8%) were male. The mean body mass index was 28.4 ± 5.1 kg/m^2^. The most prevalent comorbidities were hypertension (64.7%), chronic venous insufficiency (52.9%), and diabetes mellitus (41.2%). Six patients (17.6%) were current smokers. Wound aetiologies included venous ulcers (35.3%), diabetic ulcers (26.5%), mixed arteriovenous ulcers (17.6%), pressure injuries (11.8%), and post-surgical wounds (8.8%). The median wound duration prior to enrolment was 18 weeks (IQR 8–34). The mean baseline wound surface area was 13.2 ± 9.1 cm^2^.

**TABLE 2:** Patient demographics and baseline clinical characteristics (N = 34).

Characteristic	Value
Demographics	
Age (years), mean ± SD	62.8 ± 11.6
Male sex, n (%)	20 (58.8)
Female sex, n (%)	14 (41.2)
BMI (kg/m^2^), mean ± SD	28.4 ± 5.1
Comorbidities, n (%)	
Diabetes mellitus	14 (41.2)
Chronic venous insufficiency	18 (52.9)
Peripheral vascular disease	8 (23.5)
Hypertension	22 (64.7)
Current smoker	6 (17.6)
Immunosuppression	2 (5.9)
Wound aetiology, n (%)	
Venous	12 (35.3)
Diabetic	9 (26.5)
Mixed arteriovenous	6 (17.6)
Pressure injury	4 (11.8)
Post-surgical	3 (8.8)
Wound characteristics	
Wound duration (weeks), median (IQR)	18 (8–34)
Wound surface area (cm^2^), mean ± SD	13.2 ± 9.1
Wound surface area (cm^2^), median (range)	10.8 (2.1–42.4)
Number of wounds >1, n (%)	8 (23.5)
Wound location, n (%)	
Lower leg/ankle	15 (44.1)
Foot	10 (29.4)
Sacral/gluteal	4 (11.8)
Other	5 (14.7)

BMI, body mass index; SD, standard deviation; IQR, interquartile range.

### 3.2 Primary outcome: wound surface area reduction

**FIGURE 4:**
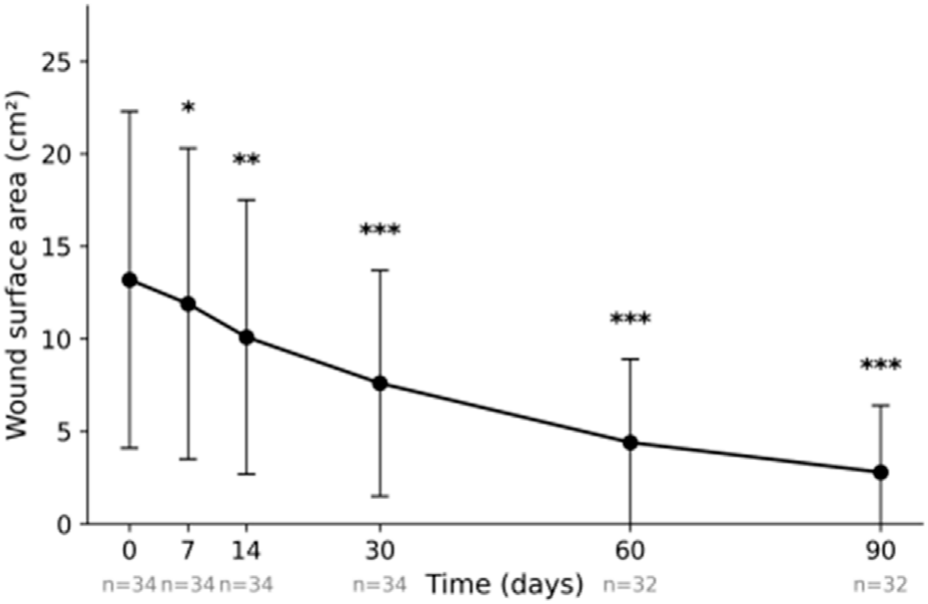
Mean wound surface area (cm^2^) over time (N = 34). Error bars represent standard deviation. *p < 0.05, **p < 0.01, ***p < 0.001 versus baseline (Wilcoxon signed-rank test).

The mean wound surface area decreased progressively from 13.2 ± 9.1 cm^2^ at baseline to 2.8 ± 3.6 cm^2^ at day 90, corresponding to a mean percentage reduction of 78.8% (95% CI, 71.4%–86.2%; p < 0.001). Statistically significant reductions from baseline were observed from day 14 onwards (Table 3; Figure 4). Because wound surface area was non-normally distributed, the Friedman test was used for the overall longitudinal comparison and confirmed a significant effect of time on wound surface area (p < 0.001), with pairwise comparisons versus baseline performed using the Wilcoxon signed-rank test. At day 30, the mean wound area reduction was 42.4%, and by day 60 it had reached 66.7%. Individual patient trajectories demonstrated consistent wound area reduction in 31 of 34 patients (91.2%); three patients showed transient wound enlargement between days 7 and 14, attributed to post-debridement inflammatory response, but all subsequently demonstrated progressive healing.

**TABLE 3:** Wound surface area and percentage reduction over time (N = 34).

Time point	WSA (cm^2^)mean ± SD	% Reductionmean ± SD	p-value vs. baseline	n
Day 0 (baseline)	13.2 ± 9.1	—	—	34
Day 7	11.9 ± 8.4	9.8 ± 8.6	0.028	34
Day 14	10.1 ± 7.4	23.5 ± 12.8	0.001	34
Day 30	7.6 ± 6.1	42.4 ± 17.2	<0.001	34
Day 60	4.4 ± 4.5	66.7 ± 18.4	<0.001	32
Day 90	2.8 ± 3.6	78.8 ± 17.8	<0.001	32

WSA, wound surface area; SD, standard deviation. p-values calculated using Wilcoxon signed-rank test. Two patients were lost to follow-up (one after day 14, one after day 30).

### 3.3 Secondary clinical outcomes

#### 3.3.1 Complete wound closure

**FIGURE 5:**
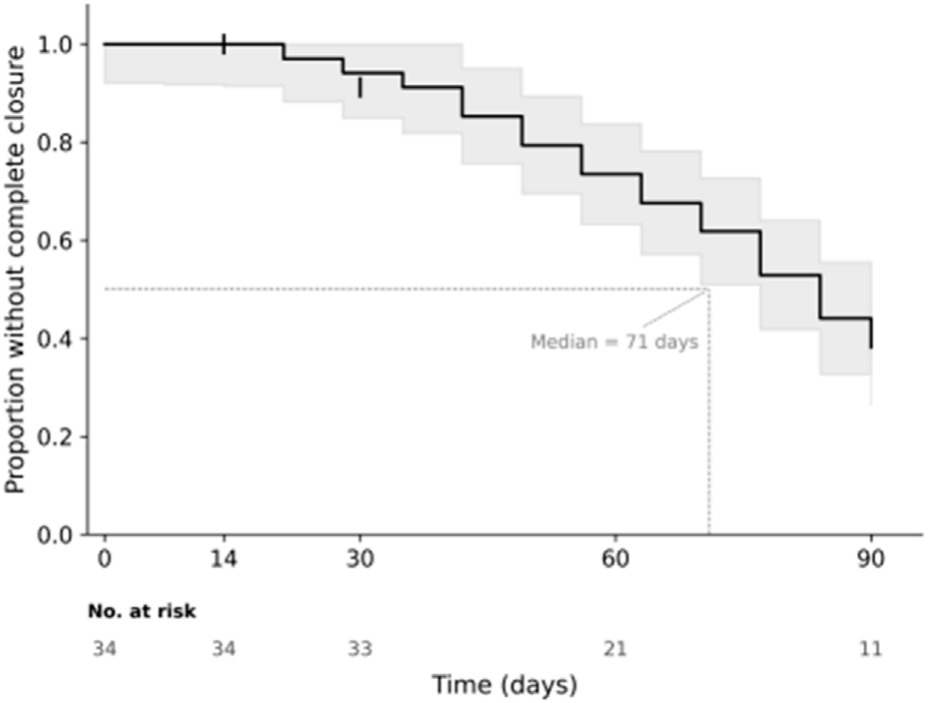
Kaplan–Meier estimate of time to complete wound closure (N = 34). Tick marks indicate censored patients. Median time to closure: 71 days (95% CI, 58–84).

Complete wound closure, defined as full epithelialization confirmed at two consecutive visits, was achieved in 21 of 34 patients (61.8%) by day 90. The cumulative closure rates were 0% at day 14, 8.8% (3/34) at day 30, 35.3% (12/34) at day 60, and 61.8% (21/34) at day 90. The median time to complete wound closure among those who healed was 71 days (95% CI, 58–84 days). Kaplan-Meier analysis is presented in Figure 5. Among the 13 patients who did not achieve complete closure by day 90, all demonstrated a wound area reduction of at least 50% (mean 61.8% ± 12.4%), and 8 of these 13 patients (61.5%) had achieved ≥75% wound area reduction by the final visit.

When stratified by wound aetiology, complete closure rates at day 90 were 75.0% (9/12) for venous ulcers, 55.6% (5/9) for diabetic ulcers, 50.0% (3/6) for mixed arteriovenous ulcers, 50.0% (2/4) for pressure injuries, and 66.7% (2/3) for post-surgical wounds. Although venous ulcers appeared to have higher closure rates, the small subgroup sizes preclude definitive comparisons.

#### 3.3.2 Wound bed tissue composition

**FIGURE 6:**
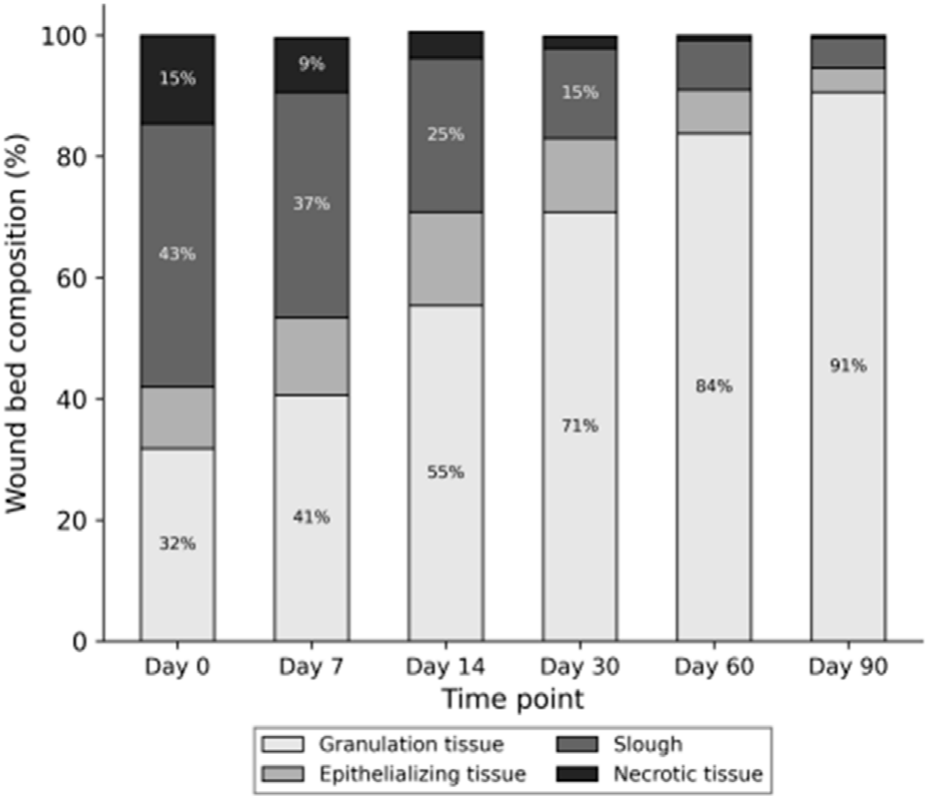
Wound bed tissue composition (%) over time, showing the proportions of granulation tissue, slough, necrotic tissue, and epithelializing tissue (N = 34).

The percentage of healthy granulation tissue in the wound bed increased progressively from 31.8% ± 18.2% at baseline to 90.6% ± 11.0% at day 90 (p < 0.001). Conversely, the percentage of slough decreased from 43.4% ± 20.6% at baseline to 5.0% ± 7.2% at day 90, and the percentage of necrotic tissue decreased from 14.6% ± 12.8% to 0.4% ± 1.0% (Table 4; Figure 6). The proportion of epithelializing tissue increased from 10.2% ± 8.6% at baseline to 46.8% ± 28.2% at day 90 in those wounds that had not yet achieved full closure. Friedman test confirmed a significant longitudinal trend for all tissue types (p < 0.001). The most rapid improvement in wound bed composition occurred between days 7 and 30.

**TABLE 4:** BWAT scores and wound bed tissue composition over time.

Parameter	Day 0	Day 7	Day 14	Day 30	Day 60	Day 90
BWAT total score						
Mean ± SD	37.8 ± 6.4	35.2 ± 6.1	31.6 ± 5.8	27.2 ± 6.3	21.8 ± 5.9	18.0 ± 6.6
p vs. baseline	—	0.018	0.002	<0.001	<0.001	<0.001
Granulation (%)						
Mean ± SD	31.8 ± 18.2	40.6 ± 17.4	55.4 ± 16.8	70.8 ± 15.6	83.8 ± 13.2	90.6 ± 1 1.0
Slough (%)						
Mean ± SD	43.4 ± 20.6	37.2 ± 18.8	25.4 ± 15.2	14.8 ± 12.8	8.2 ± 8.8	5.0 ± 7.2
Necrotic tissue (%)						
Mean ± SD	14.6 ± 12.8	9.0 ± 8.6	4.4 ± 5.0	2.0 ± 2.8	0.8 ± 1.6	0.4 ± 1.0

BWAT, Bates–Jensen wound assessment tool; SD, standard deviation. p-values calculated using Wilcoxon signed-rank test versus baseline. Day 60 and Day 90: N = 32.

#### 3.3.3 Bates–Jensen wound assessment tool scores

BWAT total scores decreased steadily from a mean of 37.8 ± 6.4 at baseline to 18.0 ± 6.6 at day 90, indicating progressive improvement in overall wound status (p < 0.001; Table 4). The greatest rate of improvement was observed between days 14 and 30. Significant improvements were noted in all individual BWAT items, with the largest magnitude of change observed in necrotic tissue amount (−1.9 ± 0.6), exudate amount (−1.7 ± 0.8), and granulation tissue (+2.1 ± 0.7) subscores.

#### 3.3.4 Wound exudate and perilesional skin

At baseline, 22 patients (64.7%) had moderate or heavy wound exudate. By day 90, only 3 of the 13 patients with open wounds (9.4% of the total cohort) had moderate exudate; no patient had heavy exudate. The proportion of patients with serous exudate increased from 32.4% at baseline to 84.6% of those with open wounds at day 90, whereas the proportion with serosanguineous or purulent exudate decreased substantially. Perilesional erythema was present in 19 patients (55.9%) at baseline and in 3 patients (9.4%) at day 90. Perilesional maceration decreased from 14 patients (41.2%) to 2 patients (6.3%), and perilesional oedema decreased from 16 patients (47.1%) to 3 patients (9.4%).

#### 3.3.5 Wound infection

Five patients (14.7%) developed clinical signs of superficial wound infection during the study period (three between days 7 and 14, and two between days 14 and 30). Wound swab cultures yielded *Staphylococcus aureus* in three cases, *Pseudomonas aeruginosa* in one, and mixed flora in one. All five infections were treated successfully with oral antibiotics (duration 7–10 days) without the need for hospitalisation. No patient developed deep wound infection, osteomyelitis, or sepsis. Three patients (8.8%) required additional surgical debridement for persistent slough (at days 18, 28, and 35, respectively); all subsequently demonstrated progressive healing.

### 3.4 Patient-reported outcome measures

#### 3.4.1 Wound pain

**FIGURE 7:**
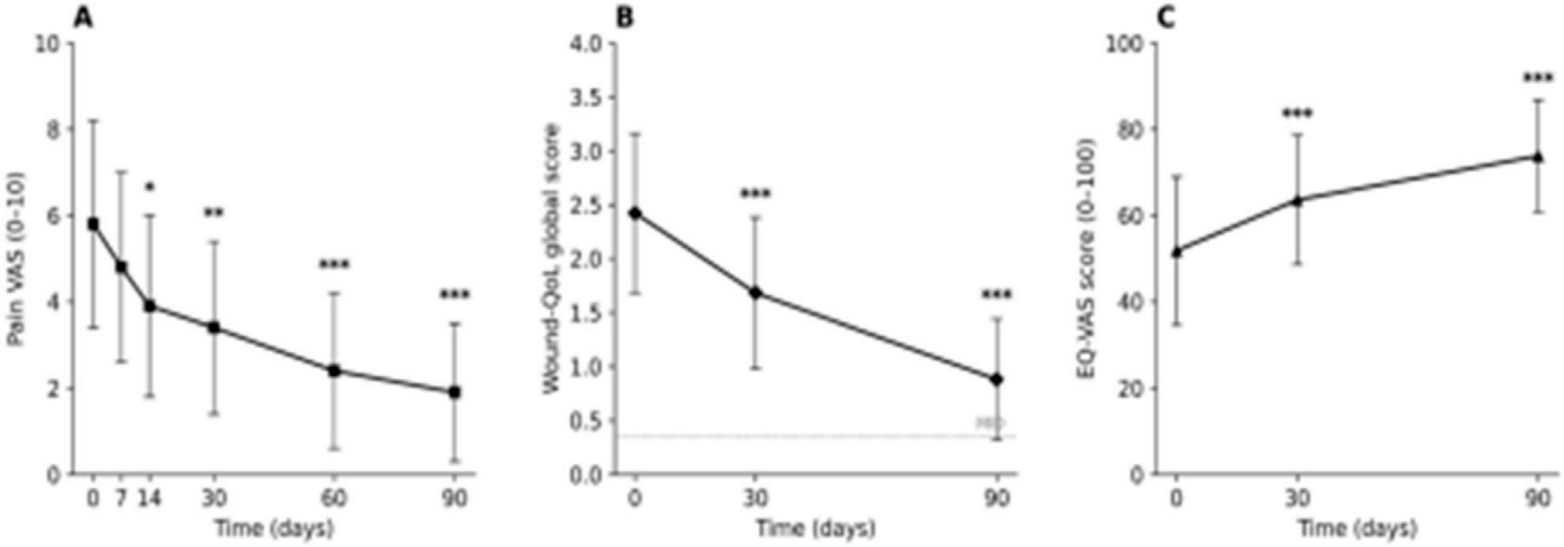
Patient-reported outcomes over time. **(A)** Mean resting pain VAS at each study visit. **(B)** Mean Wound-QoL global score at baseline, day 30, and day 90. **(C)** Mean EQ-VAS score at baseline, day 30, and day 90. Error bars represent standard deviation. ***p < 0.001 versus baseline.

Mean resting pain VAS decreased from 5.8 ± 2.4 at baseline to 1.9 ± 1.6 at day 90 (mean reduction 3.9 points; 95% CI, 3.1–4.7; p < 0.001). Pain at dressing change decreased from 6.6 ± 2.2 to 2.4 ± 1.9 (p < 0.001). The reduction in resting pain was statistically significant from day 14 onwards (Table 5). By day 90, 18 of 32 patients (56.3%) reported a resting pain VAS of ≤2. The trajectory of pain reduction is presented in Figure 7A.

**TABLE 5:** Patient-reported outcome measures at baseline, day 30, and day 90.

Outcome measure	Day 0(N = 34)	Day 30(N = 34)	Day 90(N = 32)	p-value^†^
Pain VAS (resting)				
Mean ± SD	5.8 ± 2.4	3.4 ± 2.0	1.9 ± 1.6	<0.001
Pain VAS (dressing change)				
Mean ± SD	6.6 ± 2.2	4.0 ± 2.1	2.4 ± 1.9	<0.001
Wound-QoL global score				
Mean ± SD	2.42 ± 0.74	1.68 ± 0.70	0.88 ± 0.56	<0.001
Wound-QoL – Body				
Mean ± SD	2.66 ± 0.86	1.82 ± 0.78	0.96 ± 0.64	<0.001
Wound-QoL – Psyche mean ± SD Wound-QoL – Everyday life	2.22 ± 0.80	1.46 ± 0.66	0.74 ± 0.50	<0.001
Mean ± SD	2.38 ± 0.84	1.76 ± 0.76	0.94 ± 0.60	<0.001
EQ-5D-5L index value				
Mean ± SD	0.56 ± 0.19	0.67 ± 0.17	0.78 ± 0.15	<0.001
EQ-VAS (0–100)				
Mean ± SD	51.8 ± 17.2	63.6 ± 15.0	73.8 ± 13.0	<0.001

VAS, visual analogue scale; Wound-QoL, wound quality of life questionnaire; EQ-5D-5L, EuroQol five-dimension five-level questionnaire; EQ-VAS, EQ, visual analogue scale; SD, standard deviation.

^†^ p-value for overall longitudinal trend (Friedman test).

All pairwise comparisons between baseline and day 90 were significant at p < 0.001 (Wilcoxon signed-rank test).

#### 3.4.2 Disease-specific quality of life (Wound-QoL)

The mean Wound-QoL global score decreased from 2.42 ± 0.74 at baseline to 0.88 ± 0.56 at day 90, representing a mean reduction of 1.54 points (95% CI, 1.22–1.86; p < 0.001). This change exceeds the established minimal important difference of 0.50 points (Topp et al., 2021). All three subscales showed significant improvement: body (2.66–0.96; p < 0.001), psyche (2.22–0.74; p < 0.001), and everyday life (2.38–0.94; p < 0.001). Improvements were already statistically significant at day 30 (Table 5; Figure 7B).

#### 3.4.3 Generic quality of life (EQ-5D-5L)

The mean EQ-5D-5L index value improved from 0.56 ± 0.19 at baseline to 0.78 ± 0.15 at day 90 (p < 0.001). The mean EQ-VAS score increased from 51.8 ± 17.2 to 73.8 ± 13.0 (mean improvement 22.0 points; p < 0.001; Table 5; Figure 7C). The domains demonstrating the greatest improvement were pain/discomfort and usual activities. The proportion of patients reporting moderate or worse problems in the pain/discomfort domain decreased from 82.4% at baseline to 28.1% at day 90.

### 3.5 Laboratory outcomes

#### 3.5.1 Inflammatory markers

**FIGURE 8:**
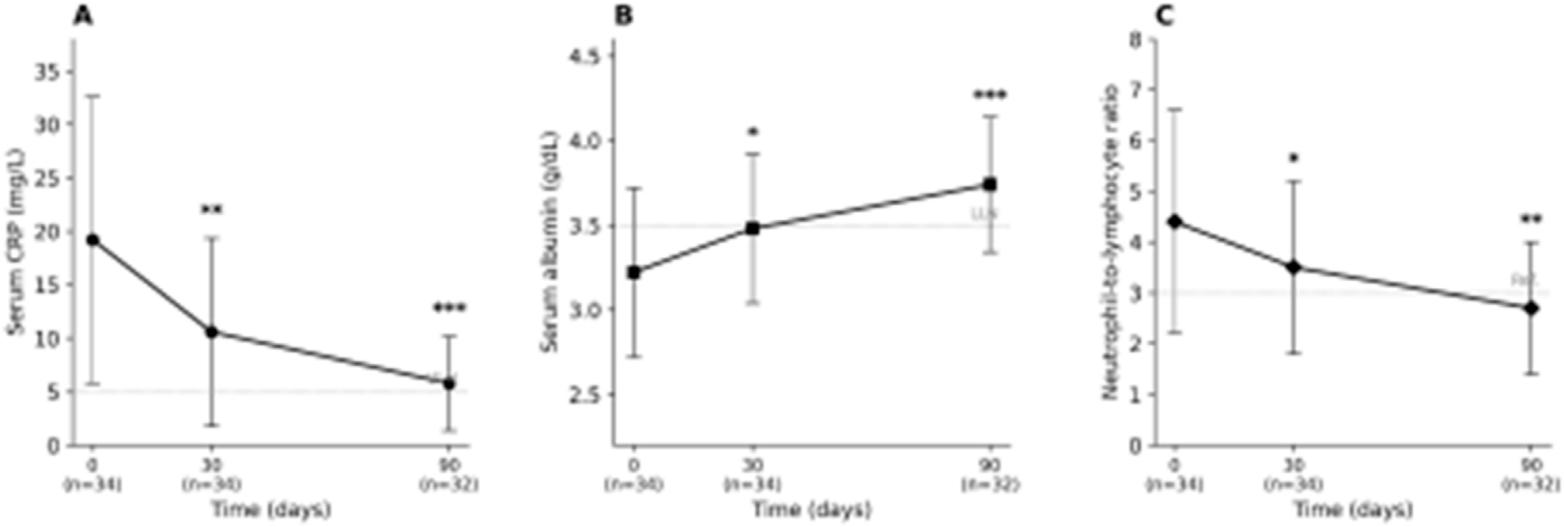
Longitudinal changes in key laboratory markers. **(A)** Serum CRP (mg/L). **(B)** Serum albumin (g/dL). **(C)** Neutrophil-to-lymphocyte ratio (NLR). Data presented as mean ± SD. *p < 0.05, **p < 0.01, ***p < 0.001 versus baseline.

Serum CRP decreased significantly from a mean of 19.2 ± 13.4 mg/L at baseline to 10.6 ± 8.8 mg/L at day 30 and 5.8 ± 4.4 mg/L at day 90 (p < 0.001; Table 6; Figure 8A). At baseline, 25 patients (73.5%) had CRP values above the upper limit of normal (≥5 mg/L); by day 90, this proportion had decreased to 11 patients (34.4%). ESR similarly decreased from 35.4 ± 19.2 mm/h to 17.0 ± 11.2 mm/h (p < 0.001). The neutrophil-to-lymphocyte ratio (NLR) decreased from 4.4 ± 2.2 at baseline to 2.7 ± 1.3 at day 90 (p = 0.002; Figure 8C), consistent with resolution of systemic inflammatory burden.

**TABLE 6:** Laboratory parameters at baseline, day 30, and day 90.

Parameter	Day 0(N = 34)	Day 30(N = 34)	Day 90(N = 32)	p-value^†^
Inflammatory markers				
CRP (mg/L)	19.2 ± 13.4	10.6 ± 8.8	5.8 ± 4.4	<0.001
ESR (mm/h)	35.4 ± 19.2	25.6 ± 14.8	17.0 ± 11.2	<0.001
NLR	4.4 ± 2.2	3.5 ± 1.7	2.7 ± 1.3	0.002
Haematological parameters				
WBC (×10^9^/L)	8.6 ± 2.8	8.0 ± 2.3	7.4 ± 1.9	0.068
Neutrophils (×10^9^/L)	5.8 ± 2.4	5.0 ± 1.9	4.4 ± 1.5	0.018
Lymphocytes (×10^9^/L)	1.5 ± 0.6	1.6 ± 0.5	1.7 ± 0.5	0.384
Haemoglobin (g/dL)	12.2 ± 1.9	12.5 ± 1.7	12.8 ± 1.5	0.148
Platelets (×10^9^/L)	264 ± 82	250 ± 66	244 ± 60	0.362
Nutritional markers				
Albumin (g/dL)	3.22 ± 0.50	3.48 ± 0.44	3.74 ± 0.40	<0.001
Prealbumin (mg/dL)	18.2 ± 5.6	21.0 ± 5.0	23.8 ± 4.4	0.001
Total protein (g/dL)	6.7 ± 0.6	6.9 ± 0.5	7.1 ± 0.4	0.162
Glycaemic control (n = 14)				
HbA1c (%)	7.9 ± 1.3	—	7.5 ± 1.1	0.168^‡^
Fasting glucose (mg/dL)	146 ± 40	136 ± 36	130 ± 34	0.198
Renal function				
Creatinine (mg/dL)	1.04 ± 0.34	1.02 ± 0.30	0.99 ± 0.28	0.648
eGFR (mL/min/1.73 m^2^)	73.8 ± 18.8	75.4 ± 17.2	77.6 ± 16.0	0.518

CRP, C-reactive protein; ESR, erythrocyte sedimentation rate; NLR, neutrophil-to-lymphocyte ratio; WBC, white blood cell count; HbA1c, glycated haemoglobin; eGFR, estimated glomerular filtration rate; SD, standard deviation. Values expressed as mean ± SD. — denotes not measured at this time point.

^†^ p-value for overall longitudinal trend (Friedman test).

^‡^ Paired t-test, day 0 vs. day 90 only (diabetic subgroup, n = 14).

#### 3.5.2 Haematological parameters

Total white blood cell count showed a non-significant trend toward reduction (8.6 ± 2.8 × 10^9^/L at baseline vs. 7.4 ± 1.9 × 10^9^/L at day 90; p = 0.068). Absolute neutrophil count decreased significantly (5.8 ± 2.4 to 4.4 ± 1.5 × 10^9^/L; p = 0.018), whereas lymphocyte count and platelet count did not change significantly. Mean haemoglobin concentration remained stable throughout the study (12.2 ± 1.9 g/dL at baseline vs. 12.8 ± 1.5 g/dL at day 90; p = 0.148; Table 6).

#### 3.5.3 Nutritional markers

Serum albumin increased significantly from 3.22 ± 0.50 g/dL at baseline to 3.74 ± 0.40 g/dL at day 90 (p < 0.001; Figure 8B). At baseline, 13 patients (38.2%) had albumin levels below 3.5 g/dL; by day 90, this proportion had decreased to 5 patients (15.6%). Serum prealbumin similarly improved from 18.2 ± 5.6 mg/dL to 23.8 ± 4.4 mg/dL (p = 0.001). Total protein remained within normal limits throughout the study and did not change significantly (p = 0.162).

#### 3.5.4 Glycaemic control and renal function

Among the 14 patients with diabetes mellitus, mean HbA1c was 7.9% ± 1.3% at baseline and 7.5% ± 1.1% at day 90 (p = 0.168). Fasting glucose showed a non-significant trend toward improvement (146 ± 40 mg/dL vs. 130 ± 34 mg/dL; p = 0.198). These findings suggest that glycaemic control remained stable throughout the study period. Serum creatinine and eGFR remained stable throughout the study (Table 6).

### 3.6 Safety outcomes

A total of 14 adverse events were recorded in 11 patients (32.4%) during the 90-day study period (Table 7). No serious adverse events (SAEs) occurred. No patient required unplanned hospitalisation related to the wound or the study intervention. No amputation was performed. Five superficial wound infections (14.7%) were treated with oral antibiotics and resolved without sequelae. Three patients (8.8%) required additional surgical debridement for persistent slough; all were considered related to the underlying wound pathology rather than to the study intervention. Donor-site complications were minor: three patients (8.8%) reported transient donor-site pain that resolved within 14 days, and two patients (5.9%) experienced delayed donor-site healing that resolved spontaneously by day 30. One patient (2.9%) developed a small wound-bed haematoma that resolved spontaneously without intervention. No cases of wound dehiscence, scaffold rejection, or allergic reaction to the collagen–elastin matrix were observed.

**TABLE 7:** Summary of adverse events (N = 34).

Adverse event	n (%)	Severity	Relation tointerve ntion	Outcome
Superficial wound infection	5 (14.7)	Mild	Unlikely	Resolved
Additional debridement required	3 (8.8)	Mild	Unlikely	Resolved
Donor-site pain	3 (8.8)	Mild	Probable	Resolved
Donor-site delayed healing	2 (5.9)	Mild	Probable	Resolved
Wound-bed haematoma	1 (2.9)	Mild	Possible	Resolved
Total adverse events	14	​	​	​
Patients with ≥1 AE, n (%)	11 (32.4)	​	​	​
Serious adverse events	0 (0)	—	—	—
Unplanned hospitalisation	0 (0)	—	—	—
Amputation	0 (0)	—	—	—

AE, adverse event. All adverse events were classified as mild in severity. No serious adverse events, unplanned hospitalisations, or amputations occurred during the study period.

### 3.7 Representative case illustrations

**FIGURE 9:**
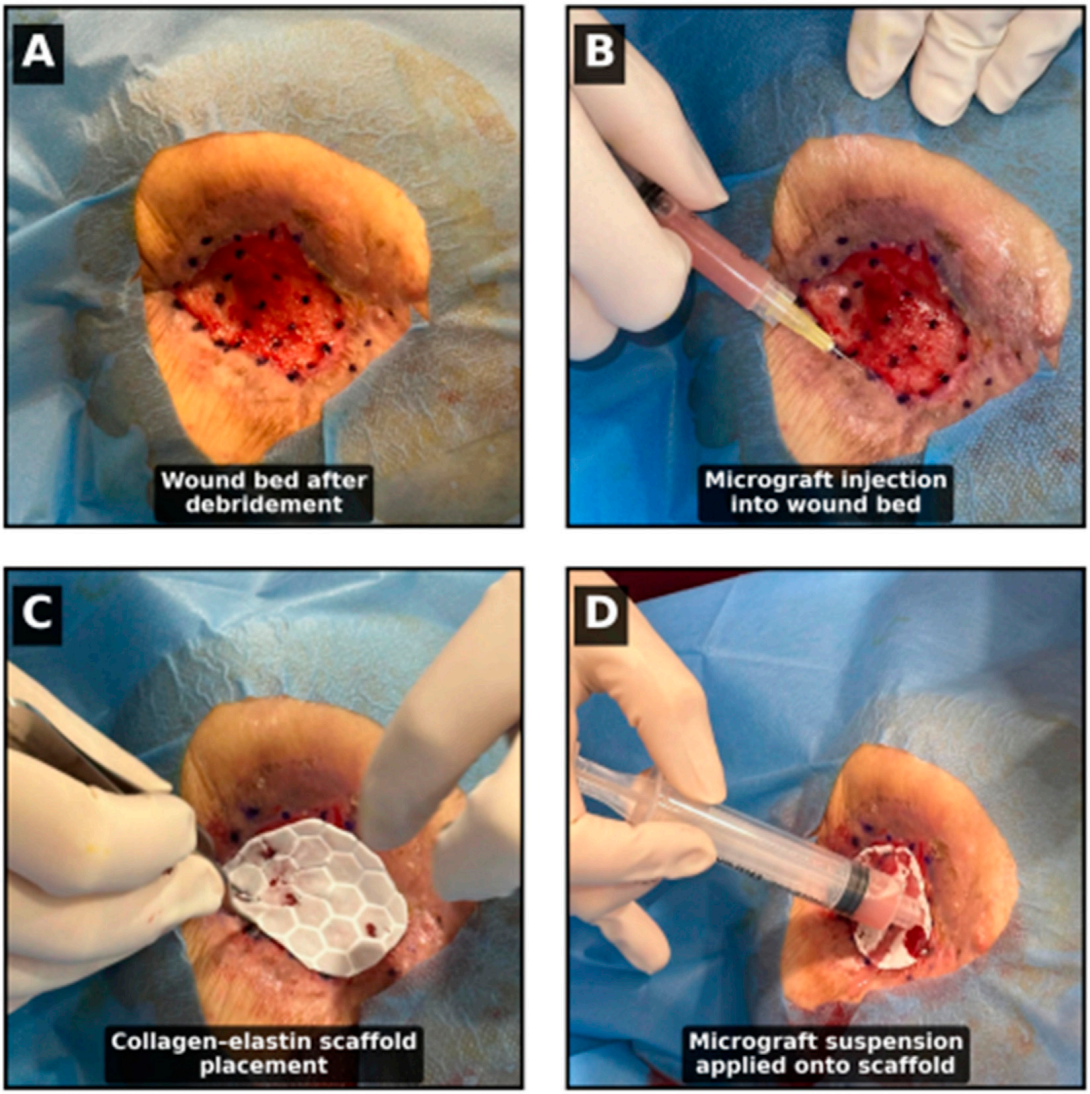
Intraoperative photographs illustrating the key steps of the combined autologous cutis and subcutis micrograft and collagen–elastin dermal scaffold procedure. **(A)** Wound bed following surgical debridement, demonstrating a well-vascularised base suitable for micrograft application. Wound margins have been marked. **(B)** Injection of the autologous cutis and subcutis micrograft suspension into the wound bed using a syringe. **(C)** Placement of the acellular collagen–elastin dermal scaffold, cut to the dimensions of the wound, onto the prepared wound bed. The characteristic honeycomb architecture of the matrix is visible. **(D)** Application of the micrograft suspension onto the dermal scaffold to imbibe the matrix with autologous cellular and extracellular material prior to final wound dressing.

**FIGURE 10:**
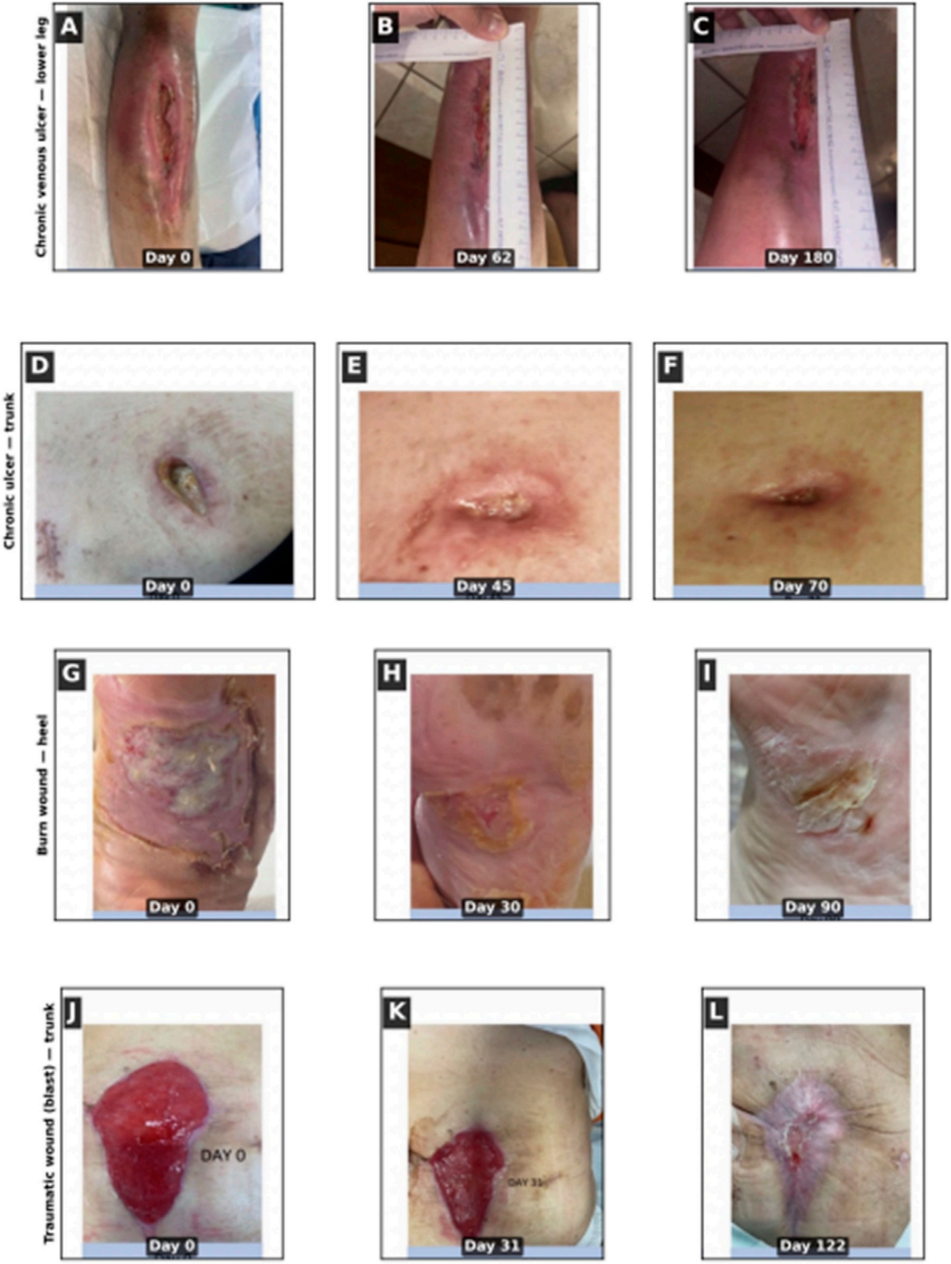
Representative serial wound photographs illustrating the clinical course in four patients treated with autologous cutis and subcutis micrografts seeded onto a collagen–elastin dermal scaffold. **(A–C)** Chronic venous ulcer of the lower leg. At baseline **(A)**, the wound presented as an elongated, full-thickness ulcer with perilesional erythema and hyperpigmentation. By Day 62 **(B)**, wound contraction and granulation tissue formation were evident. By Day 180 **(C)**, substantial wound contraction had occurred with near-complete epithelialization. **(D–F)** Chronic ulcer of the trunk. At baseline **(D)**, a deep, oval, full-thickness defect with slough at the wound base was present. By Day 45 **(E)**, granulation tissue had replaced the slough with reduction in wound depth. By Day 70 **(F)**, ongoing wound contraction and epithelialization were observed. **(G–I)** Deep partial-to-full-thickness burn of the heel. At baseline **(G)**, the wound presented with devitalised tissue and blister formation. By Day 30 **(H)**, granulation tissue formation and early epithelialization were evident. By Day 90 **(I)**, the wound had achieved substantial epithelialization with residual superficial scarring. **(J–L)** Large traumatic wound of the anterior trunk following a blast injury. At baseline **(J)**, an extensive, irregularly shaped full-thickness defect with a vascularised wound bed was present. By Day 31 **(K)**, progressive wound contraction and centripetal epithelialization were observed. By Day 122 **(L)**, near-complete wound closure had been achieved with active epithelialization and early scar formation.

Representative intraoperative photographs illustrating the key steps of the procedure are presented in Figure 9, serial wound photographs from four patients are presented in Figure 10 to illustrate the spectrum of clinical responses observed in this study.

### 3.8 Summary of key findings

In summary, treatment of chronic wounds with autologous cutis and subcutis micrografts combined with a collagen–elastin dermal scaffold was associated with a mean wound surface area reduction of 78.8% at day 90, with 61.8% of patients achieving complete wound closure. BWAT scores, wound bed tissue composition, wound exudate, and perilesional skin status all demonstrated progressive and statistically significant improvement. Patient-reported outcomes, including wound pain, disease-specific quality of life (Wound-QoL), and generic health-related quality of life (EQ-5D-5L), improved significantly, with changes exceeding established minimal important differences. Inflammatory markers (CRP, ESR, NLR) decreased significantly, and nutritional markers (albumin, prealbumin) improved, consistent with a transition from chronic inflammation toward a reparative tissue state. The intervention was well tolerated, with no serious adverse events and a favourable safety profile across all 34 treated patients.

## 4 Discussion

To the best of our knowledge, this is the first clinical study to evaluate the combination of autologous cutis and subcutis micrografts with a collagen–elastin dermal scaffold for the treatment of chronic wounds. In a cohort of 34 consecutive patients with chronic wounds refractory to conventional care, this combined regenerative approach was associated with a mean wound surface area reduction of 78.8% at day 90, with 61.8% of patients achieving complete wound closure. These results were accompanied by significant improvements in validated wound assessment scores (BWAT), wound bed tissue composition, patient-reported outcomes (pain, Wound-QoL, EQ-5D-5L), and systemic inflammatory and nutritional markers. The intervention demonstrated a favourable safety profile, with no serious adverse events.

The wound closure rate and magnitude of wound area reduction observed in this study are consistent with, and in certain respects favourable compared to, existing evidence for autologous micrograft therapies. In the pilot study by Baglioni et al., autologous micrografts applied to chronic wounds resulted in complete healing in 75% of patients by day 90 (Baglioni et al., 2024). The slightly lower complete closure rate in the present study (61.8%) may reflect differences in wound aetiology, chronicity, and baseline wound size, as well as the larger and potentially more heterogeneous patient sample. Importantly, all patients in our cohort who did not achieve complete closure nonetheless demonstrated a wound area reduction of at least 50%, and the majority achieved reductions exceeding 75%, suggesting that this combined approach facilitates progressive healing even in wounds of greater complexity.

A recent scoping review of adipose tissue micrografts and dermis micrografts in wound healing, encompassing eight clinical studies, concluded that micrograft therapy consistently demonstrated accelerated re-epithelialization, enhanced angiogenesis, improved scar remodelling, and low complication rates across diverse wound types (Zapsalis et al., 2025). Our findings are in agreement with these conclusions and extend the evidence by demonstrating that the addition of a dermal scaffold to micrograft therapy may provide a favourable structural microenvironment that supports and potentially enhances the regenerative capacity of the autologous micrografts. Consistent with this, dermis micrografts have also been applied to other complex wound types; for example, Andreone and den Hollander reported their use in combination with platelet-rich fibrin for the resurfacing of massive and chronic full-thickness burns (Andreone and den Hollander, 2019).

The collagen–elastin dermal scaffold used in this study has been independently evaluated in complex wound reconstruction, including burns and diabetic foot ulcers. Jeon et al. demonstrated that a collagen–elastin matrix produced comparable healing outcomes to split thickness skin grafting in diabetic foot ulcers, with the additional benefit of improved dermal organisation (Jeon et al., 2013). Min et al. reported that native collagen scaffolds provide guiding ridges for invading cells and may promote structured dermal wound healing (Min et al., 2021). Oehlbauer demonstrated the cost-effectiveness of collagen–elastin dermal templates in both chronic and acute wounds, suggesting that the use of such scaffolds may reduce overall treatment costs by decreasing hospitalisation time and complication rates (Oehlbauer, 2025). However, none of these studies examined the specific combination of cutis and subcutis micrografts seeded onto a collagen–elastin scaffold, which is the novel contribution of the present work.

The rationale for combining autologous cutis and subcutis micrografts with a dermal scaffold is grounded in complementary mechanisms of action. Dermis-derived mesenchymal and progenitor cells are known to promote wound healing primarily through paracrine signalling rather than direct structural contribution. They secrete a broad range of growth factors and cytokines, including vascular endothelial growth factor (VEGF), hepatocyte growth factor (HGF), transforming growth factor-β (TGF-β), and interleukin-6 (IL-6), which collectively promote angiogenesis, modulate the immune response, stimulate fibroblast and keratinocyte proliferation, and support extracellular matrix remodelling (Almalki, 2022; Hassanshahi et al., 2019). Furthermore, these cells have also been shown to promote macrophage polarisation from a pro-inflammatory (M1) to a reparative (M2) phenotype, a transition that is critical for resolving the chronic inflammatory state that characterises non-healing wounds (Lo Sicco et al., 2017; Zhang et al., 2010). Viewed collectively, this coordinated release of bioactive mediators allows the micrografts to function as a dynamic, autologous biological pharmacotherapy whose therapeutic activity arises from sustained paracrine and immunomodulatory signalling rather than from a single defined molecule. This represents a non-traditional pharmacological paradigm in which the living cellular product itself acts as both the active agent and its delivery system, continuously adapting its secretory output to the local wound microenvironment.

However, a key limitation of injectable or topically applied cell-based therapies is the rapid loss of transplanted cells from the wound bed, either through mechanical washout, cell death in the hostile wound microenvironment, or failure to engraft (Kuo et al., 2016). Dermal scaffolds may address this limitation by providing a three-dimensional structural template that retains the micrografts within the wound, protects the cellular and extracellular components from degradation, and provides physical guidance for cell migration, vascular ingrowth, and tissue organisation (Lafosse et al., 2015; Min et al., 2021).

Beyond this structural function, accumulating evidence indicates that the acellular collagen–elastin matrix exerts an active biological influence on the healing response. As the scaffold is gradually degraded by cellular proteases, its breakdown releases matrikines and elastin-derived peptides that are themselves bioactive, signalling to fibroblasts, endothelial cells, and inflammatory cells to modulate proliferation, chemotaxis, and angiogenesis. The collagen–elastin composition specifically has been shown to reduce α-smooth muscle actin expression, a marker of myofibroblast activation and fibrosis, suggesting that the elastin component may additionally temper excessive contraction and scar formation while improving the quality and elasticity of the regenerated tissue (Krymchenko et al., 2025). In this sense the scaffold is not merely an inert structural template but a bioinstructive material that participates directly in orchestrating the repair process, complementing the paracrine activity of the micrografts.

In the present study, the progressive increase in granulation tissue, the reduction in slough and necrotic tissue, and the steady improvement in BWAT scores are consistent with a transition from a chronic inflammatory wound state toward an actively proliferative and remodelling phenotype. While we cannot determine the relative contribution of the micrografts versus the scaffold from a single-arm study, the magnitude and consistency of the observed clinical improvement across wound aetiologies support the hypothesis that this combination may be synergistic.

A noteworthy finding of this study is the significant reduction in serum CRP from a mean of 19.2 mg/L at baseline to 5.8 mg/L at day 90, accompanied by parallel reductions in ESR and the neutrophil-to-lymphocyte ratio. CRP is a well-established systemic marker of inflammation, and dynamic CRP trajectories have been shown to reflect the balance between inflammatory clearance and tissue repair in chronic wounds (Jiang et al., 2026). Liu et al. demonstrated in a prospective study that serum CRP decreased significantly as chronic wounds healed, and that CRP levels correlated with wound healing trajectory (Liu et al., 2014). More recently, Wu et al. identified serum CRP as a significant predictor of 90-day wound outcomes, with an area under the receiver operating characteristic curve of 0.863 for wound CRP (Wu et al., 2025). The CRP trajectory observed in our cohort—from elevated baseline levels to near-normal values by day 90—is therefore consistent with resolution of the systemic inflammatory burden associated with chronic wound healing. The neutrophil-to-lymphocyte ratio (NLR), an inexpensive and readily available marker of systemic inflammation, decreased from 4.4 to 2.7 over the study period. While NLR has been studied extensively as a prognostic marker in oncology and cardiovascular disease, emerging evidence supports its utility in wound healing, where elevated NLR has been associated with delayed healing and poorer outcomes (Zahorec, 2021). The parallel improvement in NLR and CRP in our study suggests a systemic transition from an inflammatory to a reparative state, consistent with the clinical improvement observed at the wound level.

The significant increase in serum albumin (from 3.22 to 3.74 g/dL) and prealbumin (from 18.2 to 23.8 mg/dL) is also clinically relevant. Although traditionally interpreted as nutritional markers, albumin and prealbumin are increasingly recognised as negative acute-phase proteins whose levels are depressed by systemic inflammation rather than solely by nutritional deficiency (Kim et al., 2023; Gau et al., 2024). Thus, the improvement in these markers may reflect both the resolution of chronic inflammation and a genuine improvement in nutritional and metabolic status as the wound healing burden decreases. The proportion of patients with hypoalbuminaemia decreased from 38.2% to 15.6%, a finding with practical clinical implications, given the well-established association between low albumin and impaired wound healing.

The improvements observed in patient-reported outcomes strengthen the clinical relevance of the wound healing results. The mean reduction in resting pain VAS of 3.9 points exceeds the commonly cited minimal clinically important difference of 1.3–2.0 points for pain VAS, indicating that the pain reduction was not only statistically significant but also clinically meaningful to patients (Iglesias Lopez et al., 2023). Pain is one of the most distressing symptoms reported by patients with chronic wounds and is a major contributor to impaired quality of life, social isolation, and psychological morbidity (Augustin et al., 2014).

The improvement in disease-specific quality of life, as measured by the Wound-QoL, was substantial. The mean reduction of 1.54 points in the Wound-QoL global score considerably exceeds the minimal important difference of 0.50 points established by Topp et al. (Topp et al., 2021) This suggests that the intervention was associated with a degree of quality-of-life improvement that patients would consider highly meaningful (Topp et al., 2021). Improvements were observed across all three Wound-QoL subscales (body, psyche, and everyday life), indicating a broad positive effect on the lived experience of having a chronic wound. The generic EQ-5D-5L results corroborated these findings, with a mean improvement in index value of 0.22 and a 22-point increase in EQ-VAS, both of which exceed established thresholds for clinical importance (Herdman et al., 2011). The consistent improvement across objective clinical measures, systemic biomarkers, and patient-reported outcomes represents a triangulation of evidence that enhances the credibility of the findings from this single-arm study.

The intervention demonstrated a favourable safety profile. No serious adverse events, unplanned hospitalisations, amputations, or cases of scaffold rejection or allergic reaction were observed. The rate of superficial wound infection (14.7%) is within the expected range for chronic wounds of this complexity and was not higher than rates reported in conventional wound care populations (Frykberg and Banks, 2015). Donor-site complications were minor and self-limiting. These findings are consistent with the safety data reported in the scoping review by Zapsalis et al., which identified low complication rates across all clinical studies of cutis and subcutis micrografts (Zapsalis et al., 2025). The autologous nature of the micrografts eliminates the risk of immune rejection or disease transmission, and the point-of-care processing avoids the regulatory and logistical complexities associated with *ex vivo* culture expansion.

This study has several strengths. First, to the best of our knowledge, it is the first clinical investigation of the specific combination of autologous cutis and subcutis micrografts with a collagen–elastin dermal scaffold. Previous published studies have examined either adipose derived stromal cells seeded onto collagen matrices or dermal scaffolds used independently, but not this particular combination within a single study (Lafosse et al., 2015). Second, the study employed a comprehensive and multimodal outcome assessment framework, incorporating validated wound measurement tools (digital planimetry with dual-ruler calibration), a validated wound assessment instrument (BWAT), validated patient-reported outcome measures (VAS, Wound- QoL, EQ-5D-5L), serial systemic biomarkers, and standardised photographic documentation. This breadth of assessment is uncommon in pilot wound healing studies and provides a rich dataset for hypothesis generation (Foltynski et al., 2015; Bates-Jensen et al., 1992; Topp et al., 2021; Herdman et al., 2011). Third, the prospective, protocol-driven data collection, standardised intervention protocols, and consistent follow-up enhances the internal validity of the study.

This study has several important limitations. Most importantly, the single-arm design without a control group precludes any causal inference: the observed improvements may partly reflect the natural history of healing after thorough debridement, optimised standard wound care, the close surveillance of a study setting, or regression to the mean, and it is not possible to determine whether the outcomes exceed those achievable with standard care, micrografts alone, or the scaffold alone. External validity is a further concern, as the cohort may have comprised patients with relatively preserved healing capacity, so the single-arm results could overestimate the benefit achievable in more refractory populations. In addition, wounds of diverse aetiologies were pooled for a single efficacy analysis despite differing substantially in their underlying biology and expected healing trajectories, which limits interpretation of the aggregate outcomes and their applicability to any individual wound type.

The study was also constrained by its single-centre design, a sample size of 34 patients that—while adequate for a feasibility study—was underpowered for subgroup or robust multivariable analyses, and a 90-day follow-up that does not capture wound recurrence, durability of closure, or long-term scar quality.

Methodologically, wound bed composition was assessed by visual estimation, which remains partly subjective despite corroboration by standardised photography; complete blinding of the clinical assessor to treatment timepoint was not always feasible in a single-arm design, introducing potential assessment bias; and only systemic rather than local wound-fluid biomarkers (e.g., wound bed cytokines, matrix metalloproteinases) were collected, limiting direct insight into the local biological effects of the intervention.

The findings of this pilot study provide a foundation for several lines of future investigation. Most importantly, a randomised controlled trial comparing the combined micrograft–scaffold approach with standard wound care, or with each component alone, is needed to establish efficacy and to delineate the individual and synergistic contributions of the micrografts and the scaffold. The preliminary data and outcome measures reported here can be used to inform the sample size calculation and design of such a trial.

A multicentre study would address the generalisability limitations of the present work and would allow recruitment of a larger, more diverse patient population. Stratification by wound aetiology (venous, diabetic, pressure, mixed) in a larger cohort would enable subgroup analyses to identify which wound types derive the greatest benefit from this approach, thereby guiding patient selection in clinical practice.

From a biological standpoint, future studies should incorporate local wound fluid sampling to quantify cytokine profiles (e.g., IL-6, IL-10, TNF-α, VEGF, matrix metalloproteinases) before and after treatment, which would provide a more direct assessment of the local paracrine and immunomodulatory effects of the micrografts (Wu et al., 2025). The integration of advanced wound imaging modalities, such as laser speckle contrast imaging or hyperspectral imaging, could offer non invasive, objective assessment of wound bed perfusion and oxygenation as surrogates for angiogenesis (Kuck et al., 2022).

Extended follow-up beyond 90 days, ideally to 6 and 12 months, would enable assessment of wound recurrence rates and long-term scar quality. A cost-effectiveness analysis, conducted alongside a future controlled trial, would also be valuable given the increasing emphasis on value-based healthcare and the substantial economic burden of chronic wounds (Guest et al., 2020; Oehlbauer, 2025). Finally, the mechanistic underpinning of this combined approach warrants further investigation, including characterisation of the cellular content of the micrografts (viability, cell phenotype, growth factor content) and histological analysis of scaffold integration and dermal remodelling over time.

## 5 Conclusion

In conclusion, the combination of autologous cutis and subcutis micrografts with a collagen–elastin dermal scaffold appears to be a feasible, safe, and potentially effective regenerative approach for the treatment of chronic wounds. In this retrospective single-arm study, the intervention was associated with substantial wound area reduction, a high rate of complete wound closure, significant improvements in wound bed composition, systemic inflammatory markers, and patient-reported quality of life, with no serious adverse events. While these preliminary findings are encouraging, the absence of a control group limits causal inference, and the results should be interpreted as hypothesis-generating. Adequately powered, controlled trials—ideally with factorial designs capable of isolating the individual contributions of the micrografts and the dermal scaffold—will be needed before any efficacy claims can be established.

## Data Availability

The original contributions presented in the study are included in the article/supplementary material, further inquiries can be directed to the corresponding author.

## References

[B1] Almalki S. G. (2022). Adipose-derived mesenchymal stem cells and wound healing: potential clinical applications in wound repair. Saudi Med. J. 43 (10), 1075–1086. 10.15537/smj.2022.43.10.20220522 36261194 PMC9994497

[B2] Andreone A. den Hollander D. (2019). A retrospective study on the use of dermis micrografts in platelet-rich fibrin for the resurfacing of massive and chronic full-thickness burns. Stem Cells Int. 2019, 8636079. 10.1155/2019/8636079 31636677 PMC6766135

[B3] Augustin M. Brocatti L. K. Rustenbach S. J. Schäfer I. Herberger K. (2014). Cost-of-illness of leg ulcers in the community. Int. Wound J. 11 (3), 283–292. 10.1111/j.1742-481X.2012.01089.x 23020710 PMC7950737

[B4] Baglioni E. Trovato L. Marcarelli M. Frenello A. Bocchiotti M. A. (2016). Treatment of oncological post-surgical wound dehiscence with autologous skin micrografts. Anticancer Res. 36 (3), 975–980. 26976986

[B5] Baglioni E. A. Perego F. Paolin E. Abate A. Pusceddu T. Zavan B. (2024). Efficacy of autologous micrografts technology: a promising approach for chronic wound healing and tissue regeneration—a pilot study. Front. Med. (Lausanne) 11, 1417920. 10.3389/fmed.2024.1417920 39131083 PMC11310043

[B6] Bates-Jensen B. M. Vredevoe D. L. Brecht M. L. (1992). Validity and reliability of the pressure sore status tool. Decubitus 5 (6), 20–28. 1489512

[B7] Bates-Jensen B. M. McCreath H. E. Harputlu D. Patlan A. (2019). Reliability of the Bates-Jensen wound assessment tool (BWAT) for pressure injury assessment: the pressure Ulcer detection study. Wound Repair Regen. 27 (4), 386–395. 10.1111/wrr.12714 30828890 PMC6693585

[B8] Blome C. Baade K. Debus E. S. Price P. Augustin M. (2014). The “Wound-QoL”: a short questionnaire measuring quality of life in patients with chronic wounds based on three established disease-specific instruments. Wound Repair Regen. 22 (4), 504–514. 10.1111/wrr.12193 24899053

[B9] Cervero-Ferragut S. Martinez-Santamaria L. Guerrero-Aspizua S. (2017). Quantitative analysis of blood cells and inflammatory factors in wounds. J. Tissue Viability 26 (1), 58–62. 10.12968/jowc.2017.26.3.121

[B10] De Francesco F. Graziano A. Trovato L. Ceccarelli G. Romano M. Marcarelli M. (2017). A regenerative approach with dermal micrografts in the treatment of chronic ulcers. Stem Cell Rev. Rep. 13 (1), 139–148. 10.1007/s12015-016-9692-2 27738884

[B11] Enoch S. Grey J. E. Harding K. G. (2004). Should alternative endpoints be considered to evaluate outcomes in chronic recalcitrant wounds? World Wide Wounds. Available online at: http://www.worldwidewounds.com/2004/october/Enoch-Part2/Alternative-Enpoints-To-Healing.html.

[B12] Foltynski P. Ladyzynski P. Ciechanowska A. Migalska-Musial K. Judzewicz G. Sabalinska S. (2015). Wound area measurement with digital planimetry: improved accuracy and precision with calibration based on 2 rulers. PLoS One 10 (8), e0134622. 10.1371/journal.pone.0134622 26252747 PMC4529141

[B13] Frykberg R. G. Banks J. (2015). Challenges in the treatment of chronic wounds. Adv. Wound Care New Rochelle. 4 (9), 560–582. 10.1089/wound.2015.0635 26339534 PMC4528992

[B14] Gau B. R. Chen H. Y. Hung S. Y. (2024). Examining albumin as a bioindicator of healing capability in patients with diabetic foot ulcers: a retrospective review. Wounds 36 (3), 86–92. 37347595

[B15] Guest J. F. Fuller G. W. Vowden P. (2020). Cohort study evaluating the burden of wounds to the UK’s National Health Service in 2017/2018: update from 2012/2013. BMJ Open 10, e045253. 10.1136/bmjopen-2020-045253 33371051 PMC7757484

[B16] Hassanshahi A. Hassanshahi M. Khabbazi S. Hosseini-Khah Z. Peymanfar Y. Ghalamkari S. (2019). Adipose-derived stem cells for wound healing. J. Cell Physiol. 234 (6), 7903–7914. 10.1002/jcp.27922 30515810

[B17] Herber O. R. Schnepp W. Rieger M. A. (2007). A systematic review on the impact of leg ulceration on patients’ quality of life. Health Qual. Life Outcomes 5, 44. 10.1186/1477-7525-5-44 17651490 PMC1947954

[B18] Herdman M. Gudex C. Lloyd A. Janssen M. Kind P. Parkin D. (2011). Development and preliminary testing of the new five-level version of EQ-5D (EQ-5D-5L). Qual. Life Res. 20 (10), 1727–1736. 10.1007/s11136-011-9903-x 21479777 PMC3220807

[B19] Iglesias Lopez R. A. Gonzalez Fernandez M. L. Heredia E. A. Mosquera Fernandez A. (2023). Pain assessment in patients with chronic wounds: a visual analogue scale approach. Int. Wound J. 20 (4), 1098–1111.36181308

[B20] International Wound Infection Institute (IWII) (2022). Wound Infection in Clinical Practice: Principles of Best Practice. London: Wounds International.

[B21] Jeon H. Kim J. Yeo H. Jeong H. Son D. Han K. (2013). Treatment of diabetic foot ulcer using a collagen-elastin matrix in comparison with a skin graft. Arch. Plast. Surg. 40 (4), 403–408. 10.5999/aps.2013.40.4.403 23898439 PMC3724003

[B22] Jiang S. Song Q. Wang J. Sun X. Zhang Z. Wang S. (2026). Dynamic C-reactive protein trajectories predict prolonged healing time in diabetic wounds: a machine learning model based on a single-center cohort with standardized wound size. Front. Med. (Lausanne) 13, 1778003. 10.3389/fmed.2026.1778003 41767538 PMC12935653

[B23] Kim K. K. Jang C. S. Eun S. C. (2023). Nutritional markers for predicting lower extremity free tissue transfer outcomes in the chronic wound population. Microsurgery 43 (1), 30–37. 10.1002/micr.30794 34357655

[B24] Krymchenko R. Avila-Martinez N. Gansevoort M. Bakker G. J. Gomes M. L. N. P. Vlig M. (2025). Collagen-elastin dermal scaffolds enhance tissue regeneration and reduce scarring in preclinical models. Mater Today Bio 34, 102239. 10.1016/j.mtbio.2025.102239 40917518 PMC12408406

[B25] Kuck M. Schoenborn K. H. Sterry W. Lademann J. Darvin M. E. (2022). Heterogeneous perfusion patterns in chronic wounds revealed by hyperspectral imaging. Wound Repair Regen. 30 (5), 614–625.

[B26] Kuo Y. R. Wang C. T. Cheng J. T. Kao G. S. Chiang Y. C. Wang C. J. (2016). Adipose-derived stem cells accelerate diabetic wound healing through the induction of autocrine and paracrine effects. Cell Transpl. 25 (1), 71–81. 10.3727/096368915X687921 25853951

[B27] Lafosse A. Desmet C. Aouassar N. André W. Hanet M. S. Beauloye C. (2015). Autologous adipose stromal cells seeded onto a human collagen matrix for dermal regeneration in chronic wounds: clinical proof of concept. Plast. Reconstr. Surg. 136 (2), 279–295. 10.1097/PRS.0000000000001437 25946602

[B28] Liu F. Yang Z. Li C. Yi X. Bai X. (2014). A prospective pilot study to evaluate wound outcomes and levels of serum C-reactive protein and interleukin-6 in the wound fluid of patients with trauma-related chronic wounds. Ostomy Wound manage. 60 (6), 30–37. 24905355

[B29] Lo Sicco C. Reverberi D. Balbi C. Ulivi V. Principi E. Pascucci L. (2017). Mesenchymal stem cell-derived extracellular vesicles as mediators of anti-inflammatory effects: endorsement of macrophage polarization. Stem Cells Transl. Med. 6 (3), 1018–1028. 10.1002/sctm.16-0363 28186708 PMC5442783

[B30] Marcarelli M. Trovato L. Novarese E. Riccio M. Graziano A. (2017). Rigenera protocol in the treatment of surgical wound dehiscence. Int. Wound J. 14 (1), 277–281. 10.1111/iwj.12601 27126653 PMC7949493

[B31] Min S. Yoon J. Y. Park S. Y. (2021). Biological dermal templates with native collagen scaffolds provide guiding ridges for invading cells and may promote structured dermal wound healing. J. Tissue Viability 30 (3), 403–413. 10.1111/iwj.13314 PMC7949003 32045112

[B32] Oehlbauer M. (2025). Cost-effectiveness of native collagen-elastin dermal regeneration template in chronic and acute wounds. J. Burn Care Res. 46 (Suppl. 1). S336. 10.1093/jbcr/iraf019.444

[B33] Sbitan A. Al-Qudah O. Chbib C. Camargo C. P. (2025). Adipose tissue and fat-derived products in wound, ulcer, and scar management: a systematic review. Front. Surg. 12, 1666776. 10.3389/fsurg.2025.1666776 41141701 PMC12546055

[B34] Topp J. Blome C. Augustin M. Mohr N. Debus E. S. Diener H. (2021). Determining the minimal important difference for the Wound-QoL questionnaire. Patient Prefer Adherence 15, 1571–1578. 10.2147/PPA.S315822 34285475 PMC8286720

[B35] Tresoldi M. M. Graziano A. Malovini A. Faga A. Nicoletti G. (2019). The role of autologous dermal micrografts in regenerative surgery: a clinical experimental study. Stem Cells Int. 2019, 9843407. 10.1155/2019/9843407 31582991 PMC6754962

[B36] von Elm E. Altman D. G. Egger M. Pocock S. J. Gotzsche P. C. Vandenbroucke J. P. (2007). The strengthening the reporting of observational studies in epidemiology (STROBE) statement: guidelines for reporting observational studies. Lancet 370 (9596), 1453–1457. 10.1016/s0140-6736(07)61602-x 18064739

[B37] Wendelken M. E. Berg W. T. Lichtenstein P. Alvarez O. M. Markowitz L. Comfort C. (2011). Wounds measured from digital photographs using photodigital planimetry software: validation and rater reliability. Wounds 23 (9), 267–275. 25879267

[B38] World Medical Association (2013). World medical association declaration of Helsinki: ethical principles for medical research involving human subjects. JAMA 310 (20), 2191–2194. 10.1001/jama.2013.281053 24141714

[B39] Wu Y. C. Chen Y. L. Lai W. F. (2025). Promising biomarkers for chronic wound healing: a pilot cohort study on wound cytokines and a novel biofilm detection kit for predicting 90-day outcomes. Wound Repair Regen. 33 (1), e70100. 10.1111/wrr.70100 41074722

[B40] Zahorec R. (2021). Neutrophil-to-lymphocyte ratio, past, present, and future perspectives. Bratisl. Lek. Listy 122 (7), 474–488. 10.4149/bll_2021_078 34161115

[B41] Zapsalis K. Ioannidis O. Anestiadou E. Pantelidou M. Siozos K. Xylas C. (2025). Applications of adipose tissue micrografts (ATM) and dermis micrografts (DMG) in wound healing: a scoping review of clinical studies. Bioeng. (Basel) 12 (9), 948. 10.3390/bioengineering12090948 41007193 PMC12467152

[B42] Zhang Q. Z. Su W. R. Shi S. H. Wilder-Smith P. Xiang A. P. Wong A. (2010). Human gingiva-derived mesenchymal stem cells elicit polarization of M2 macrophages and enhance cutaneous wound healing. Stem Cells 28 (10), 1856–1868. 10.1002/stem.503 20734355 PMC3114043

